# Non-antibiotic pharmaceuticals promote the transmission of multidrug resistance plasmids through intra- and intergenera conjugation

**DOI:** 10.1038/s41396-021-00945-7

**Published:** 2021-03-10

**Authors:** Yue Wang, Ji Lu, Shuai Zhang, Jie Li, Likai Mao, Zhiguo Yuan, Philip L. Bond, Jianhua Guo

**Affiliations:** Advanced Water Management Centre, The University of Queensland, St. Lucia, Brisbane, QLD Australia

**Keywords:** Antibiotics, Public health

## Abstract

Antibiotic resistance is a global threat to public health. The use of antibiotics at sub-inhibitory concentrations has been recognized as an important factor in disseminating antibiotic resistance via horizontal gene transfer. Although non-antibiotic, human-targeted pharmaceuticals are widely used by society (95% of the pharmaceuticals market), the potential contribution to the spread of antibiotic resistance is not clear. Here, we report that commonly consumed, non-antibiotic pharmaceuticals, including nonsteroidal anti-inflammatories (ibuprofen, naproxen, diclofenac), a lipid-lowering drug (gemfibrozil), and a *β*-blocker (propranolol), at clinically and environmentally relevant concentrations, significantly accelerated the dissemination of antibiotic resistance via plasmid-borne bacterial conjugation. Various indicators were used to study the bacterial response to these drugs, including monitoring reactive oxygen species (ROS) and cell membrane permeability by flow cytometry, cell arrangement, and whole-genome RNA and protein sequencing. Enhanced conjugation correlated well with increased production of ROS and cell membrane permeability. Additionally, these non-antibiotic pharmaceuticals induced responses similar to those detected when bacteria are exposed to antibiotics, such as inducing the SOS response and enhancing efflux pumps. The findings advance understanding of the transfer of antibiotic resistance genes, emphasizing the concern that non-antibiotic, human-targeted pharmaceuticals enhance the spread of antibiotic resistance among bacterial populations.

## Introduction

Increasingly, antimicrobial resistance is a major threat to public health, causing 700,000 deaths worldwide each year [1]. Bacterial antibiotic resistance mainly occurs through a mutation in DNA or by acquiring antibiotic resistance genes (ARGs) through horizontal gene transfer (HGT) [2, 3]. HGT consists of three different pathways: conjugation, transformation and transduction. Among them, conjugation is the primary mechanism for disseminating antibiotic resistance [4]. During conjugation, the exchange of genetic material between the donor and recipient occurs through direct cell-to-cell contact or via a connecting pilus [5]. Typically, the exchange is mediated by mobile genetic elements, such as a conjugative plasmid.

It is commonly accepted that the emergence and spread of antibiotic resistance is largely due to intensive applications of antibiotics in clinical, veterinary, and agricultural settings [6]. Exposure of microorganisms to antibiotics that are below the minimal inhibitory concentration (MIC) can promote HGT [7, 8]. For example, the antibiotics aminoglycoside and fluoroquinolone were shown to induce genetic transformability in the pathogen *Streptococcus pneumoniae* [7]. Although the consumption of non-antibiotic pharmaceuticals occupies approximately 95% of the drug market [9, 10], the role of these pharmaceuticals in the emergence and spread of antibiotic resistance has received relatively little attention. Recently, Maier et al. [11] screened more than 1,000 marketed drugs against 40 representative gut bacterial strains, and reported that more than 200 non-antibiotic pharmaceuticals could exhibit antibiotic-like effects on the bacteria. The authors found these non-antibiotic pharmaceuticals contributed to the emergence of antibiotic resistance through increased expression of efflux pump genes [11]. We previously showed that several commonly consumed non-antibiotic pharmaceuticals (e.g., non-steroidal anti-inflammatory drugs) could facilitate the spread of antibiotic resistance via natural transformation [12]. This previous study demonstrated that for a single population (i.e., naturally competent bacterium *Acinetobacter baylyi*), the transfer of free plasmids could be enhanced by non-antibiotic pharmaceuticals. However, it remains unknown whether non-antibiotic pharmaceuticals promote conjugation between two intra- or intergenera populations, which is of particular clinical concern as conjugative multidrug resistance plasmids allow rapid expression of multidrug resistance phenotypes, thus facilitating the emergence and spread of antibiotic-resistant bacteria [13]. In addition, it is not clear whether there are common features or properties of non-antibiotic pharmaceuticals, or shared mechanisms, that promote the horizontal transfer of ARGs.

In this study, we aimed to investigate the potential of different types of commonly consumed non-antibiotic, human-targeted pharmaceuticals for promoting conjugative transfer of plasmid-borne ARGs. To this end, we established two conjugative models consisting of both intragenera and intergenera conjugations, in which ARGs were harbored on environmentally relevant conjugative plasmid RP4 [14], or clinically relevant broad-host-range pMS6198A [15]. We applied culture-based methods to calculate conjugative plasmid transfer ratio. The underlying mechanisms were revealed using a combination of phenotypic testing (culturing experiments and fluorescence-based flow cytometry) and genotypic testing (plasmid electrophoresis, whole-genome RNA sequencing and proteomic analysis). The pharmaceuticals tested included nonsteroidal anti-inflammatory drugs (NSAIDs) (ibuprofen, naproxen, diclofenac), a lipid-lowering drug (gemfibrozil), a *β*-blocker (propranolol), and a contrast medium (iopromide). These drugs are used in a wide range of clinical settings including for pain/fever-relief, treatment of inflammation, lipid control, heart disease, and diagnostic medicine. All these pharmaceuticals are on the World Health Organization List of Essential Medicines, and are widely consumed. For example, worldwide there are 30 million users of NSAIDs daily, and over 100 million consumers of NSAIDs annually in the USA alone [16]. Once consumed, such drugs present in the human gut or plasma at high concentrations [17–19]. In addition, a large portion of the drug (e.g., up to 90%) is excreted unchanged in the urine, destined for wastewater and other environments [20–22]. Thus, these pharmaceuticals are recognized as emerging contaminants and are ubiquitously detected in various environments, including wastewater, surface water, groundwater, and even drinking water, ranging in concentrations from nanograms to milligrams per litre [23, 24].

## Materials and methods

### Bacterial strains and MIC determination

Two conjugation models were applied in this study, i.e., environmentally relevant Model-1, and clinically relevant Model-2. In Model-1, *Escherichia coli* K-12 LE392 with plasmid RP4 (resistant to tetracycline, kanamycin and ampicillin) was the donor [25]. *Pseudomonas putida* KT2440 (i.e., *Pseudomonas alloputida* [26]) with high resistance to chloramphenicol was used as the recipient [27, 28]. *E. coli* MG1655 with pMS6198A (a *bla*_NDM-1_-positive IncA/C plasmid, isolated from a multidrug-resistant uropathogenic *E. coli* strain with resistance to drugs of last resort, including carbapenem), was the donor for Model-2 [15]. *E. coli* J53 with resistance to sodium azide was the recipient for Model-2 [15]. Culture conditions are described in Text S1.

Bacterial MICs for antibiotics and non-antibiotic pharmaceuticals were determined according to previous methods [27, 29]. MICs were calculated based on the comparison between pharmaceutical-dosed groups and either sterilized MilliQ water, ethanol, or dimethyl sulfoxide (DMSO). Details are described in Text S2.

### Environmentally and clinically relevant conjugative transfer with the addition of non-antibiotic pharmaceuticals under aerobic conditions

This study established two mating models to investigate if non-antibiotic pharmaceuticals could promote gene transfer. In the environmentally relevant conjugation model (Model-1), donor and recipient both at a concentration of 10^8^ cfu/mL were mixed 1:1 to establish the PBS-based conjugative mating system (pH=7.2), using a total volume of 1 mL. Various concentrations of non-antibiotic pharmaceuticals were added to the mating system, including clinically and environmentally relevant concentrations, and sub-MIC levels, i.e., 0.005, 0.05, 0.5, 5, 50 mg/L for ibuprofen, naproxen, gemfibrozil, diclofenac, propranolol, and 0.01, 0.1, 1, 5, 50 mg/L for iopromide [17–19, 21, 24]. After 8 h-incubation at 25 °C without shaking, 50 *μ*L of the mixture was spread onto LB agar selection plates containing antibiotics to enumerate transconjugants, with details described in Text S3.

For Model-2, donor and recipient bacterial strains were both grown to an OD600nm value of 1.8, and mixed 1:2 in LB broth according to a previous study [15]. The non-antibiotic pharmaceuticals were dosed as per those in the environmentally relevant conjugation. After 2 h-incubation at 37 °C under static conditions, 20 μL of the mixture was plated on to antibiotic selective plates to enumerate transconjugants, with details shown in Text S3.

In addition to the above matings, further sets of Model-1 were established with the addition of 100 μM ROS scavenger, thiourea. The conjugative transfer ratio was calculated from the number of transconjugant colonies divided by the number of recipients. As no nutrients were provided during the mating process, the growth of donor, recipient, and transconjugant was neglected.

To test the reverse transfer process, transconjugants obtained from Model-1 were used as the new donor, while a mutant strain of *E. coli* MG1655 with chloramphenicol resistance was the recipient [30]. The conjugation experiments were conducted with various non-antibiotic pharmaceuticals, as described above. Transconjugants were enumerated on Difco^TM^ m Endo agar plates (to distinguish *E. coli* and *P. putida*) with the appropriate antibiotics, as described in Text S3.

The conjugative plasmids applied in this study are both large, i.e., 60.09 kb for RP4, and 137.57 kb for pMS6198A, and none of the recipient bacterial strains were competent cells. Thus, the bacterial transformation was ruled out [31].

### Plasmid verification

Model-1 transconjugants growing on selective plates were randomly picked, cultured, and stored in 25% glycerol at −80 °C. The plasmids of transconjugants were extracted using the Invitrogen PureLink Quick Plasmid Miniprep Kit (Life Technologies, USA). The specific *traF* gene of plasmid RP4 was amplified by PCR, and the amplicons were observed using 1% agarose gel electrophoresis. To further verify the identity of the plasmid, PCR was applied for detection of the *tetA* and *bla*_TEM_ from the RP4 plasmid. Similarly, the presence of pMS6198A in transconjugants from Model-2 was determined by genome DNA extraction followed by PCR using primers for *bla*_NDM_. PCR primers and conditions are described in Text S4 and Supplementary Table S1.

### Transmission electron microscopy (TEM)

TEM (JEOL JEM-1011, Japan) operated at 80 kV was used to observe the effect of pharmaceuticals on bacterial cells. Conjugation experiments were performed as described above, and TEM samples were collected after 8-h mating with either 0.5 mg/L ibuprofen, naproxen, gemfibrozil, diclofenac, propranolol, or 1.0 mg/L iopromide. Sample preparations were performed according to standard procedures as previously described [32], and details are illustrated in Text S5.

### ROS generation and cell membrane permeability

The fluorescence method was used to analyse ROS generation and cell membrane permeability, as described in Text S6. ROS-generating bacterial cells were stained with 2’, 7’-dichlorofluorescein diacetate (DCFDA) dye, and bacteria cells with a permeable membrane were stained with propidium iodide (PI). After exposure to the various non-antibiotic pharmaceuticals, 20 μM of DCFDA and 2 mM of PI were applied to the donor and recipient cells. The dyed cells were then detected using a CytoFLEX S flow cytometer (Beckman Coulter, USA). In addition, a ROS scavenger, thiourea, was also added in pharmaceutical-exposed bacterial cells, to detect the effect of thiourea on ROS generation and cell membrane permeability. The DCFDA- and PI- stained cells were recorded and calculated as fold changes compared to the control group (absence of added pharmaceuticals).

### Conjugative transfer and ROS generation under anaerobic conditions

To further verify whether ROS is crucial to the conjugation process, conjugation Model-1 and ROS generation were additionally conducted under anaerobic conditions. The assays were the same as that under aerobic conditions, except that oxygen in LB or PBS was depleted and the experiments were conducted in an anaerobic chamber (Coy Laboratory Products Inc., USA).

### Whole-genome RNA sequence analysis and bioinformatics

In order to analyze the gene expression levels during the conjugative process, conjugation experiments (Model-1) were performed as described above, and RNA was extracted after 2-h mating with either 0.5 mg/L ibuprofen, naproxen, gemfibrozil, diclofenac, propranolol, or 1.0 mg/L iopromide. As bacterial mRNA expression responds quickly to external stress, a 2-h mating time was chosen, as per previously [28, 33]. Total RNA (containing the mixture of donor and recipient bacteria) was extracted using RNeasy Mini Kit (QIAGEN^®^, Germany), with an extra bead-beating step for cell lysis [27]. Triplicate RNA samples were then submitted to Macrogen Co. (Seoul, Korea) for strand specific cDNA library construction and Illumina paired-end sequencing (HiSeq 2500, Illumina Inc., San Diego, CA). Raw data were analyzed using the bioinformatics pipeline described previously [32]. The database used for alignment was a combination of the reference genome of *E. coli* K-12 (NC_000913), *P. putida* KT2440 (NC_002947), and IncP*α* RP4 plasmid (L27758), obtained from the National Center for Biotechnology Information (NCBI). Regarding the bioinformatics pipeline, NGS QC Toolkit (v2.3.3) [34], SeqAlto (version 0.5) [35], and Cufflinks (version 2.2.1) [36] were applied to treat the raw sequence reads, and to analyze the differential expression for triplicate samples. CummeRbund package in R was used to conduct the statistical analyses [36, 37]. The measure of ‘fragments per kilobase of a gene per million mapped reads’ (FPKM) was used to quantify gene expression. Gene expression between the control (no added pharmaceuticals) and the pharmaceutical-exposed groups were compared. In this study, a cut-off of log_2_ fold-change ≥1.0 or ≤ −1.0 with both *P* value and false discovery rate (*q* value) < 0.05 was used to distinguish the differentially expressed genes.

### Proteomic analysis and bioinformatics

Conjugation experiments (Model-1) were established as described above to compare proteins expressed in the donor and recipient bacteria during the absence and presence of the non-antibiotic pharmaceuticals. Initially, the optimal length of exposure period was examined for the conjugations when exposed to either 0.5 mg/L gemfibrozil or propranolol. Total proteins from the mixture of donor and recipient bacteria were extracted after 2, 4, 6, and 8 h mating as described previously [27]. For peptide preparations, the extracted proteins were treated by reduction, alkylation, trypsin digestion, and ziptip clean-up procedures as described previously [38]. The peptides were then analysed by mass spectrometry (Triple-T 5600 (ABSciex, USA), equipped with a Nanospray III interface). Qualitative protein libraries were constructed by information dependent analysis, while quantitative protein determination was based on SWATH-MS [38] using triplicate samples. Database and software analyses and settings were performed as described in Text S7. A stringency cut-off of *q* value less than 0.01 was used to identify the proteins with significantly different expression levels. Based on the number of proteins showing significant variations, 8 h was selected as the best exposure time for the proteomic analysis. Thus, another set of conjugation experiments was established as described above using the 8 h mating period in the presence of either ibuprofen, naproxen, gemfibrozil, diclofenac, or propranolol, each at 0.5 mg/L, or with iopromide at 1.0 mg/L. Following that, for each of the conjugation experiments, the proteins were extracted, peptides prepared, and proteomic analyses were performed as described above.

### Correlation tests

Correlation tests were conducted to identify whether the phenotypic data (including conjugative transfer ratio, ROS generation and cell membrane permeability) were concentration-dependent. Linear regressions were performed for log-transformed concentration and the corresponding phenotypic data. A concentration-dependence was seen when *R*^2^ > 0.9. Further, Pearson correlation was applied to calculate correlation coefficient *r*, which was significant if *P*-value was less than 0.05.

### Statistical analysis

Data were expressed as mean ± standard deviation (SD). SPSS for Mac version 25.0 was applied for data analysis. Independent-sample *t-*tests were performed and Benjamini–Hochberg correction method was applied for multiple comparisons [39]. *P-*values less than 0.05 were considered to be statistically significant. All the experiments were conducted in triplicate.

## Results

### Non-antibiotic pharmaceuticals significantly accelerate the conjugative transfer of ARGs

**Fig. 1:**
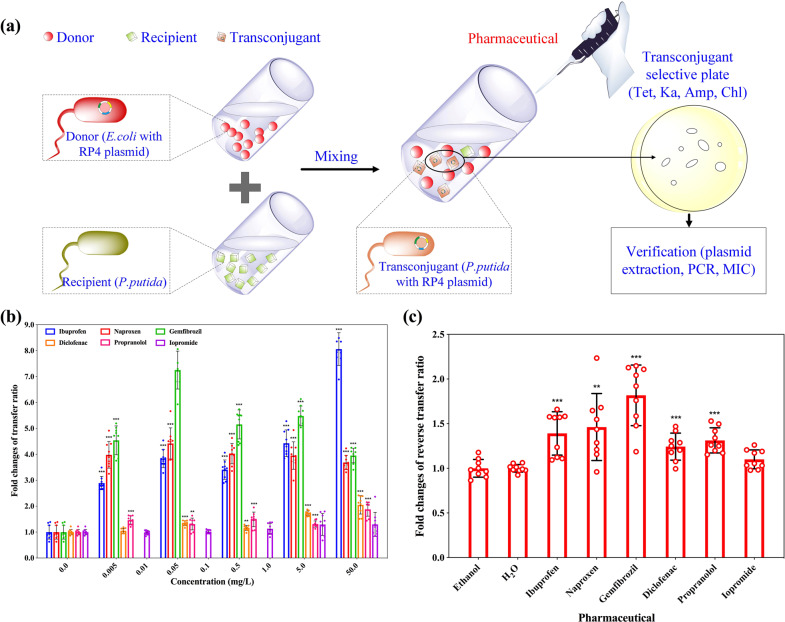
Effects of non-antibiotic pharmaceuticals on the conjugative transfer of ARGs. **a** Schematic experimental design of the conjugation. **b** Fold changes of transfer ratio under the exposure of non-antibiotic pharmaceuticals, the histogram bars with the same colour refer to the same pharmaceutical. **c** Fold changes of reverse transfer ratio under the exposure of non-antibiotic pharmaceuticals (0.5 mg/L for ibuprofen, naproxen, gemfibrozil, diclofenac, propranolol, and 1.0 mg/L for iopromide). Significant differences between non-antibiotic-dosed samples and the control were analyzed by independent-sample *t* test and corrected by Benjamini–Hochberg method for multiple comparisons, *P < 0.05, **P < 0.01, and ***P < 0.001.

To evaluate the effects of six non-antibiotic pharmaceuticals on environmentally relevant conjugation, we used *E. coli* LE392 as the donor with the conjugative RP4 plasmid harboring multiple resistance genes against tetracycline, kanamycin, and ampicillin. *Pseudomonas putida* KT2440, with high tolerance towards chloramphenicol, was the recipient in Model-1 [27]. During the conjugation process the cells were exposed to sub-inhibitory, non-antibiotic pharmaceuticals (MICs of pharmaceuticals are shown in Supplementary Table S2), at concentrations from 0.005 to 50 mg/L (both clinically and environmentally relevant concentrations were included [17–19, 23, 24]) to test if they would increase the transfer of ARGs. After the cross-genera mating, transconjugants were enumerated on plates containing four antibiotics (tetracycline, kanamycin, ampicillin and chloramphenicol). The transfer events in different treatment groups were enumerated as the absolute number of transconjugants, and normalized as the transfer ratio, which was calculated as the number of transconjugants divided by the number of recipients (Fig. 1a).

For both the number of transconjugants and the transfer ratio, it was found that for the addition of non-antibiotic pharmaceuticals, ibuprofen, naproxen and gemfibrozil, at all five concentrations (from 0.005 to 50 mg/L), the fold change increase in the absolute number of transconjugants was significant (*P* = 1 × 10^−8^–6 × 10^−4^) (Supplementary Fig. S1). For diclofenac and propranolol, only the higher concentrations (5 or 50 mg/L) increased transconjugant number. In contrast, none of the applied iopromide concentrations increased the transconjugant number. Using ibuprofen as a specific example, the fold change of transconjugant absolute number (compared with no ibuprofen dosage) increased from 2.8 ± 0.2- to 7.3 ± 0.8-fold when increasing its dosage from 0.005 mg/L to 50 mg/L. All non-antibiotic pharmaceuticals, except for iopromide, at concentrations as low as 0.05 mg/L, significantly increased conjugative transfer ratios (*P* = 3 × 10^−8^–0.017) (Fig. 1b, Supplementary Fig. S1). The increase was as high as 8-fold when exposed to 50 mg/L ibuprofen for 8 h. Even low concentrations (0.005 mg/L) of ibuprofen, naproxen and gemfibrozil showed significant enhancement in the conjugative ratio (*P* = 5 × 10^−8^–3 × 10^−6^). It should be noted that ibuprofen, naproxen and gemfibrozil were dissolved in ethanol due to their limited solubility in water. In order to rule out the effect caused by the solvent, another set of conjugation experiments without any solvent was established with the dosage of ibuprofen (0.005 mg/L, 0.05 mg/L), naproxen (0.005 mg/L, 0.05 mg/L) and gemfibrozil (0.005 mg/L, 0.05 mg/L), which were the highest concentrations that can be dissolved in MilliQ water. A significant increase in both absolute transconjugant number and transfer ratio were also observed (Supplementary Table S3), indicating ibuprofen, naproxen and gemfibrozil increased the conjugative transfer of ARGs, regardless of the solvent. In addition, we observed that gemfibrozil of 0.5 or 5 mg/L, diclofenac of 5 or 50 mg/L, and propranolol of 0.5, 5, 50 mg/L could exhibit significant decrease on total viable recipient cell numbers. In contrast, other concentrations of all other pharmaceuticals did not show significant decrease on total viable recipient cell numbers. Moreover, no significant correlations were seen between cell death and conjugative transfer ratio (Supplementary Table S4).

To verify the successful transfer of the RP4 plasmid, gel electrophoresis showed that the plasmids in transconjugants were the same as that in the donor, while no plasmid was seen in the recipient (Supplementary Fig. S1). The specific primers generated three bands on gel electrophoresis. PCR of *tetA* and *bla*_TEM_ genes (both short and long primers applied) also indicated the transconjugant plasmids harbored the donor genes (Supplementary Fig. S1). Transconjugant MICs for the four antibiotics, tetracycline, kanamycin, ampicillin, and chloramphenicol, were the same as those of the donor and recipient bacteria (Supplementary Table S5).

We also found that the RP4 plasmid was able to transfer from the transconjugant to the recipient bacterium *E. coli* MG1655 [30]. In addition, when exposing the reverse mating system to ibuprofen, naproxen, gemfibrozil, diclofenac, and propranolol at 0.5 mg/L, the respective transfer ratio fold change significantly increased (*P* = 4 × 10^−7^–0.003) (Fig. 1c).

In order to rule out that the RP4 plasmid confers a selective advantage to bacteria exposed to these non-antibiotic pharmaceuticals, growth curves of the randomly selected transconjugants and recipients were compared under various pharmaceutical concentrations [27]. Maximum growth rates of transconjugants and their corresponding recipient at the same pharmaceutical dosage were compared. The results suggested that the RP4 plasmid did not confer any significant selective advantage to the bacteria exposed to these non-antibiotic pharmaceuticals (*P* = 0.052–0.989) (Supplementary Table S6).

Moreover, this study adopted a clinically relevant bacterial conjugation model (i.e., Model-2) to validate whether the non-antibiotic pharmaceuticals could also promote conjugative transfer, in which *E. coli* MG1655 harboring conjugative plasmid pMS6198A was the donor, and *E. coli* J53 with resistance to sodium azide was the recipient [15]. Similarly, the addition of non-antibiotic pharmaceuticals (except iopromide) significantly increased both the absolute number of transconjugants and transfer ratio (*P* = 2 × 10^−7^–0.035) (Supplementary Fig. S2). Compared with the solvent control (DMSO for all six pharmaceuticals), changes in transconjugant number and transfer ratio ranged from 1.4 ± 0.2- to 2.7 ± 0.3-fold (*P* = 2 × 10^−5^–0.003), and 1.6 ± 0.2- to 3.4 ± 0.7-fold (*P* = 6 × 10^−6^–0.005), respectively. In contrast, iopromide did not significantly change the clinically relevant conjugation (*P* = 0.051–0.109). The successful transfer of pMS6198A was verified by PCR of inherent *bla*_NDM_ (Supplementary Fig. S2). Total viable recipient cell number did not decrease significantly under the exposure to these non-antibiotic pharmaceuticals (except exposed to 0.5 or 50 mg/L naproxen, Supplementary Table S7). Therefore, this clinically relevant conjugation model further validated that non-antibiotic pharmaceuticals (except iopromide) promote the conjugative transfer of plasmid-borne antibiotic resistance.

It should be noted that ethanol was used to dissolve ibuprofen, naproxen, and gemfibrozil due to their limited solubility in water. However, we can rule out the effects of ethanol on the conjugative process for the following reasons: (i) The transconjugant number and the calculated conjugation ratio were compared with their corresponding solvent control; (ii) In the environmentally relevant conjugation model (Model-1), dose-response effects were observed for these three pharmaceuticals dissolved in ethanol. The increased conjugative ratio with the elevated level of non-antibiotic pharmaceuticals indicated that pharmaceuticals themselves were enhancing the conjugation, rather than quenching the effects of solvent; (iii) When these three pharmaceuticals were directly dissolved in MilliQ water at maximal concentration, concentrations of 0.005 and 0.05 mg/L still promoted conjugation; and (iv) In the clinically relevant conjugation model (Model-2), where all the non-antibiotic pharmaceuticals were dissolved in DMSO, five pharmaceuticals enhanced the conjugation in comparison with the DMSO control group. Thus, it was confirmed that the non-antibiotic pharmaceuticals promoted conjugative transfer of ARGs, regardless of the solvent.

Collectively, it was concluded that non-antibiotic pharmaceuticals (excepting for iopromide) at environmentally and clinically relevant concentrations, significantly increased conjugative transfer of multiresistance genes (*P* = 1×10^−8^–0.038). In addition, the transconjugant was able to transfer the plasmid containing multidrug resistance genes, becoming a new source of ARGs.

### ROS play a role in enhancing conjugative transfer

**Fig. 2:**
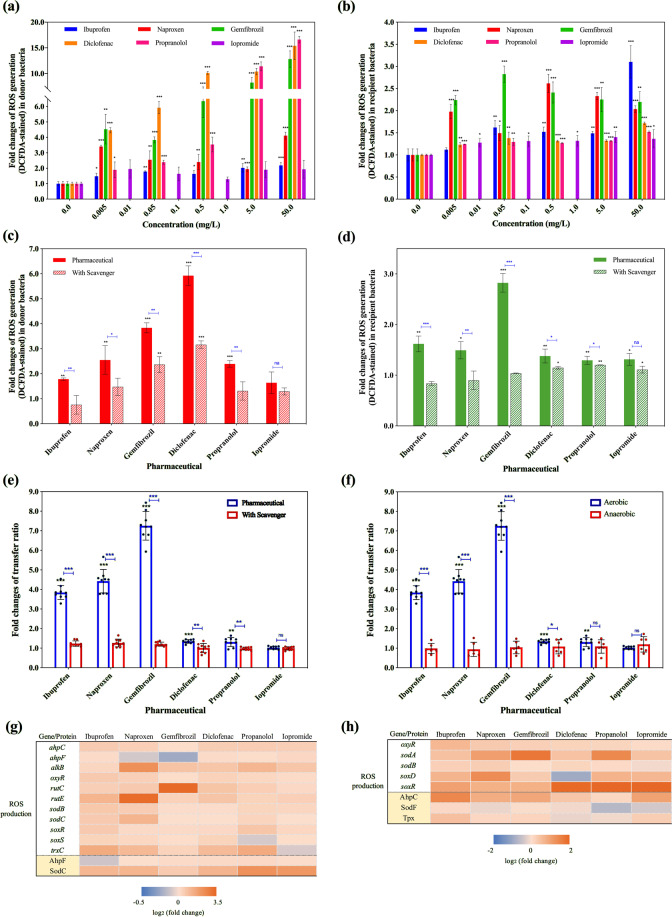
Effects of non-antibiotic pharmaceuticals on ROS in the donor (*E. coli* K-12 LE392) and recipient (*P. putida* KT2440) bacteria. **a** Fold changes of ROS generation (DCFDA-stained) in donor bacteria, the histogram bars with the same colour refer to the same pharmaceutical. **b** Fold changes of ROS generation (DCFDA-stained) in recipient bacteria, the histogram bars with the same colour refer to the same pharmaceutical. **c** Fold changes of ROS generation (DCFDA-stained) in donor bacteria with the addition of ROS scavenger thiourea. **d** Fold changes of ROS generation (DCFDA-stained) in recipient bacteria with the addition of ROS scavenger thiourea. **e** Fold changes of conjugative transfer ratio with the addition of ROS scavenger thiourea. **f** Fold changes of conjugative transfer ratio under aerobic and anaerobic conditions. **g** Fold changes of expression of core genes and proteins related to ROS production in donor bacteria. **h** Fold changes of expression of core genes and proteins related to ROS production in recipient bacteria. Significant differences between non-antibiotic-dosed samples and the control were analyzed by independent-sample *t* test and corrected by Benjamini–Hochberg method for multiple comparisons, *P < 0.05, **P < 0.01, and ***P < 0.001. For **c**–**f**, figures shown are 0.05 mg/L for ibuprofen, naproxen, gemfibrozil, diclofenac, propranolol, and 0.1 mg/L for iopromide. For **g**–**h**, figures shown are 0.5 mg/L for ibuprofen, naproxen, gemfibrozil, diclofenac, propranolol, and 1.0 mg/L for iopromide.

ROS are natural byproducts of bacterial metabolism. However, under environmental stress, ROS production may increase dramatically, and this may enhance conjugative transfer [25, 27]. In conjugation experiments described above, the fluorescence-measured ROS production was seen to increase significantly in both the donor and recipient under exposure to the five non-antibiotic pharmaceuticals (except for iopromide) (*P* = 2 × 10^−7^–0.041) (Supplementary Fig. S3). In comparison to the corresponding control group, the donor bacteria ROS levels increased from 2 ± 0.1-fold, to up 15 ± 0.5-fold at exposure to 50 mg/L propranolol (Fig. 2a). The fold changes in ROS generation in the recipient were relatively lower than those in the donor, where the highest change was 3 ± 0.3-fold with exposure at 50 mg/L ibuprofen (Fig. 2b). Moreover, the effects of diclofenac and propranolol on ROS generation in the donor were concentration-dependent (*R*^2^ = 0.94, *r* = 0.84, *P* < 0.05 for diclofenac, and *R*^2^ = 0.91, *r* = 0.86, *P* < 0.01 for propranolol), and higher ROS levels were detected with increasing concentrations of pharmaceuticals. In contrast, the effects of ibuprofen, naproxen and gemfibrozil on ROS were not concentration dependent (*P* > 0.05). This indicated that these non-antibiotic pharmaceuticals at concentrations as low as 0.005 mg/L increased ROS generation, albeit in a non-linear trend. It should also be noted that the ethanol solvent did not increase ROS generation.

ROS scavenger, thiourea, significantly decreased the effect of non-antibiotic pharmaceuticals in both donor and recipient bacteria (*P* = 1 × 10^−6^–0.045, Fig. 2c, d). The effects of ROS on the conjugation process were reversed by adding thiourea during the mating period. As illustrated in Fig. 2e, the conjugative transfer ratio declined significantly for all the pharmaceuticals (except iopromide) (*P* = 5 × 10^−5^–0.007) in the presence of the scavenger. For example, with 0.05 mg/L gemfibrozil and naproxen, the transfer ratio decreased from 7 ± 0.7-fold and 4 ± 0.6-fold to only 1.2 ± 0.1-fold and 1.3 ± 0.2-fold, respectively, when the scavenger was added. In addition, no significant increase was observed in the transfer ratio between the controls (no drug) and the scavenger-dosed drug groups (*P* = 0.052–0.94). It should be noted that no significant variations of ROS generation or transfer ratio were observed between the non-pharmaceutical control groups with and without thiourea dosage. These indicated that ROS scavenger thiourea itself did not affect ROS generation or conjugative transfer ratio.

To further verify whether ROS is crucial for the enhanced conjugation, we detected both ROS generation and conjugative transfer under anaerobic conditions. It was found that no ROS was generated anaerobically, which was consistent with the previous study [40]. Meanwhile, the transfer ratio decreased to the non-pharmaceutical control level (Fig. 2f). This further confirmed that ROS is playing a key role in the enhanced conjugation.

Moreover, in the conjugation experiments, expression levels of RNA and protein were compared between the non-antibiotic pharmaceutical-dosed groups and the control groups (without pharmaceuticals) of the donor and recipient bacteria. This was conducted to further understand the effects of these pharmaceuticals on conjugation. It was seen that pharmaceuticals significantly upregulated ROS production-related proteins and genes in both donor and recipient (Fig. 2g, h, Supplementary Tables S8–S11). For the donor bacterium, these pharmaceuticals increased expression of redox-sensing genes, *oxyR* and *soxR*, which are regulators of genes defending against oxidative stress [41, 42] (Fig. 2g). Proteins responsible for alkyl hydroperoxide reductase (AhpF) and superoxide dismutase (SodC) activities increased significantly with the dosage of pharmaceuticals (*q* < 0.01). For example, expression of SodC was enhanced 4.7 ± 0.6-fold when exposed to 0.5 mg/L propranolol. Correspondingly, genes coding for hydroperoxide reductase (*ahpC* and *ahpF*), oxidative demethylase (*alkB*), superoxide dismutase (*sodB* and *sodC*) and superoxide response (*soxS*) increased by 1.1 ± 0.3- to 4.8 ± 0.9-fold with exposure to pharmaceuticals. These genes are involved in the bacterial response to high-level oxidative stress [43–45]. Notably, iopromide of 1.0 mg/L had the least effect on ROS-related gene expression levels in the donor bacterium, which is in agreement with lower levels of ROS generation (Fig. 2a). For the recipient bacterium, these non-antibiotic pharmaceuticals increased abundances of alkyl hydroperoxide reductase (AhpF) and hydroperoxide peroxidase (Tpx), but only ibuprofen and gemfibrozil enhanced the expression of superoxide dismutase protein (SodF) (Fig. 2h). Additionally, the expression of redox-sensing gene, *oxyR*, and superoxide dismutase regulators, *sodA* and *sodB*, was significantly enhanced with exposure to each of the non-antibiotic pharmaceuticals.

### Cell membrane variations are linked to increased conjugation

**Fig. 3:**
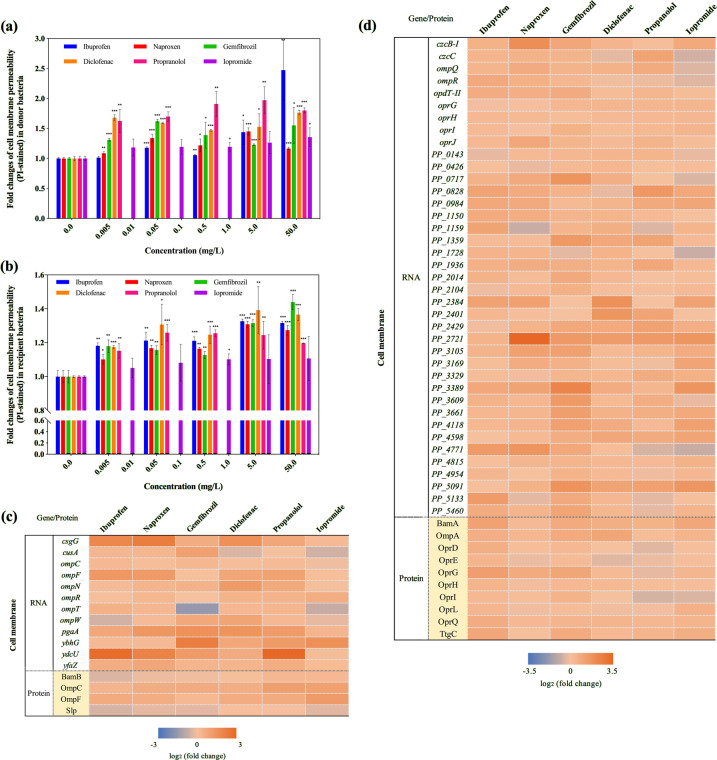
Effects of non-antibiotic pharmaceuticals on cell membranes in the donor (*E. coli* K-12 LE392) and the recipient (*P. putida* KT2440) bacteria. **a** Fold changes of cell membrane permeability (PI-stained) in donor bacteria, the histogram bars with the same colour refer to the same pharmaceutical. **b** Fold changes of cell membrane permeability (PI-stained) in recipient bacteria, the histogram bars with the same colour refer to the same pharmaceutical. **c** Fold changes of expression of core genes and proteins related to cell membranes in donor bacteria. **d** Fold changes of expression of core genes and proteins related to cell membranes in recipient bacteria. Significant differences between non-antibiotic-dosed samples and the control were analyzed by independent-sample *t* test and corrected by Benjamini–Hochberg method for multiple comparisons, *P < 0.05, **P < 0.01, and ***P < 0.001. For **c** and **d**, figures shown are 0.5 mg/L for ibuprofen, naproxen, gemfibrozil, diclofenac, propranolol, and 1.0 mg/L for iopromide.

Cell membranes are barriers during the conjugative process [46, 47]. Speculating that non-antibiotic pharmaceuticals may increase conjugative transfer by affecting the bacterial cell membrane, we tested cell membrane permeability by flow cytometry in the presence and absence of pharmaceuticals. For the donor bacteria, naproxen, gemfibrozil, diclofenac, and propranolol at the low concentration of 0.005 mg/L were seen to increase the cell membrane permeability significantly (*P* = 6 × 10^−6^–0.037) (Fig. 3a and Supplementary Fig. S4). Ibuprofen at concentrations higher than 0.05 mg/L significantly increased the membrane permeability (*P* = 0.001–0.028), while iopromide had no effect (*P* = 0.041–0.107) (Fig. 3a). The impact of ibuprofen on the donor bacterial cell membrane permeability increased with increasing ibuprofen concentration, and a 2.5 ± 0.4-fold change was detected at 50.0 mg/L. In contrast, for the other non-antibiotic pharmaceuticals, the membrane permeability changes were not concentration-dependent (*P* > 0.05). The results matched well with the conjugative transfer changes detected, where the ratio increased with increasing ibuprofen concentrations (Fig. 1b). For the recipient bacteria, all the chosen concentrations of ibuprofen, naproxen, gemfibrozil, diclofenac, and propranolol significantly enhanced the membrane permeability (*P* = 3 × 10^−7^–0.02) (Fig. 3b and Supplementary Fig. S4). These increases in cell membrane permeability were likely contributing to the increased conjugation observed in the presence of these non-antibiotic pharmaceuticals. We also observed that the addition of ROS scavenger (thiourea) could eliminate the pharmaceutical-enhanced cell membrane permeability in donor bacteria (Supplementary Fig. S4). Meanwhile, for the non-pharmaceutical control, dosing thiourea did not change the cell membrane permeability. These results suggested that thiourea itself did not have any impacts on cell membrane, and the permeable cell membrane should be resulted from the elevated ROS level.

Using TEM, we examined the effect of the pharmaceuticals on cell morphology and arrangement during the conjugation periods. During exposure to the pharmaceuticals (except iopromide) cell membranes were partially damaged, and the cell arrangement became more compact with cells drawn closer to each other (Supplementary Fig. S5). In contrast, for iopromide, the cells remained intact and separated (Supplementary Fig. S5). The effects of non-antibiotic pharmaceuticals on cell arrangement were further demonstrated by the decreased distances between adjacent cells, which were measured by ImageJ software. The average distance between cells in the control group was calculated to be 0.49 *μ*m (*n* = 100 for each group), while the addition of non-antibiotic pharmaceuticals (except iopromide) decreased the distance to 0.23–0.32 *μ*m (*P* = 2 × 10^−5^) (Supplementary Fig. S5). Iopromide did not cause any significant decrease in inter-cellular distance compared to the control (0.45 μm, *P* = 0.38). During the conjugation process direct donor and recipient cell contact is necessary for plasmid transfer [48]. Thus, the closer cell contact and membrane damage detected here aligns with the changes in membrane permeability, and the correspondingly higher levels of gene transfer detected in the presence of the pharmaceuticals. This provides further explanation for the enhanced conjugative transfer detected for ibuprofen, naproxen, gemfibrozil, diclofenac and propranolol, and is in agreement with the lack of effect by iopromide.

Moreover, the variations in cell membrane permeability induced by non-antibiotic pharmaceuticals were supported by the analyses of both RNA and protein. Core genes and proteins related to cell membrane structure and function showed significant changes with exposure to non-antibiotic pharmaceuticals (Supplementary Tables S12–S15). Regulator proteins, which alter the levels of outer membrane channels and membrane permeability [49, 50], increased significantly after exposure to the non-antibiotic pharmaceuticals (*q* < 0.01). For example, the abundance of OmpC and OmpF in the donor bacteria, and OmpA, OprH, OprL and OprQ in the recipient bacteria, significantly increased (up to 2.4 ± 0.6-fold) in all of the five pharmaceutical-dosed groups (Fig. 3c, d). The correspondingly relevant genes also showed significantly increased expression, including *ompC*, *ompF*, *ompN*, *ompR* in the donor bacteria, and *oprG*, *oprH*, *oprI*, *oprJ* in the recipient bacteria. Notably, the expression of *ompC*, *ompF*, *ompN* in the donor bacteria were unchanged for iopromide, while the other five pharmaceuticals caused up to 2.5 ± 0.7-fold change. A decrease in expression of *ompQ* and *ompR* was detected in the recipient bacteria after dosing iopromide, whereas ibuprofen, naproxen, gemfibrozil, diclofenac and propranolol caused increased expression from 1.3 ± 0.2- to 1.8 ± 0.3-fold. These variations also partially explain the different effects of pharmaceuticals on the conjugation process. In addition, putative genes which code for outer membrane proteins in donor bacteria [51], also increased significantly with exposure to non-antibiotic pharmaceuticals. For example, the expression of genes *csgG*, *cusA*, *pgaA*, *ybhG*, *ydcU*, *yfaZ* increased by up to 8-fold (iopromide exposure showed the least increase), and these may also contribute to the increased cell membrane permeability.

### Other key factors regulating the conjugative process

Genes on the conjugative plasmid are also key factors in regulating conjugation, which involves the coordinated processes of replication, partitioning and conjugation [52]. For the RP4 plasmid, important plasmid borne factors for the conjugative process are those involving DNA-transfer replication and mating pair formation [53].

**Fig. 4:**
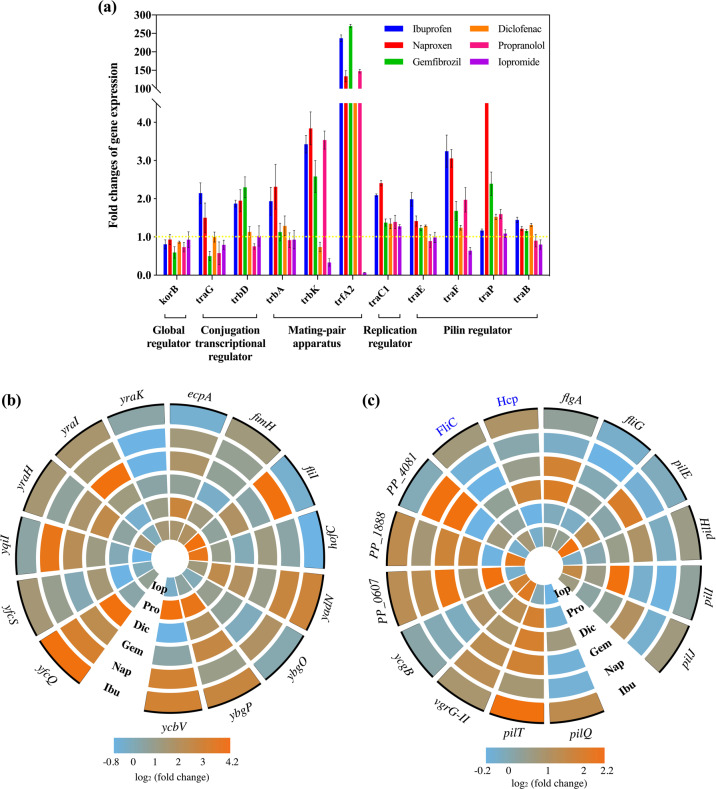
Effects of non-antibiotic pharmaceuticals on fimbriae gene expression in the donor (*E. coli* K-12 LE392), recipient (*P. putida* KT2440) bacteria, and core gene expression in conjugative plasmid (IncP-α RP4 plasmid). **a** Fold changes of expression of core genes in RP4 plasmid. **b** Fold changes of expression of core genes related to fimbriae in donor bacteria. **c** Fold changes of expression of core genes and proteins related to fimbriae in recipient bacteria. Ibu, Nap, Gem, Dic, Pro, and Iop refer to 0.5 mg/L ibuprofen, 0.5 mg/L naproxen, 0.5 mg/L gemfibrozil, 0.5 mg/L diclofenac, 0.5 mg/L propranolol, and 1.0 mg/L iopromide, respectively.

In particular, the global regulator *korB* alters operon expression of the IncP-*α* RP4 plasmid. With exposure to these non-antibiotic pharmaceuticals the expression of *korB* was repressed up to 1.7 ± 0.2-fold decrease (Fig. 4a), thus, leading to the enhanced expression of genes for the mating pair apparatus, replication and conjugative regulators. For example, ibuprofen at 0.5 mg/L caused the enhanced expression of the conjugative transfer transcriptional regulators, *traG* and *trbD* by up to 2.2 ± 0.2- and 1.9 ± 0.1-fold, respectively, and caused up-regulation of the mating pair apparatus, including *trbA*, *trbK*, *trfA2*, by up to 237 ± 7.5-fold. Ibuprofen also increased expression of the replication regulator, with a 2.1 ± 0.1-fold change in *traC1* detected. Similar changes were seen when the RP4 plasmid was exposed to naproxen, gemfibrozil, diclofenac, and propranolol. Notably, iopromide had the least effect on *korB* expression, with a 1.1 ± 0.2-fold decrease, thus, having a lower effect on other core genes in the RP4 plasmid. For example, expression of *trfA2*, which is responsible for mating pair formation and replication in the RP4 plasmid [54, 55], decreased by 10 ± 2-fold with iopromide, which partially explains why iopromide was less effective in promoting the conjugal process. However, the expression of *trfA2* was enhanced by 56 ± 3.8- to 270 ± 3.9-fold when exposed to the other five pharmaceuticals (Supplementary Table S16).

During the conjugation process, the plasmid is transferred through a pilin bridge, and the pilin-related genes in RP4 plasmid include *traB*, *traE*, *traF*, and *traP* [56]. With exposure to ibuprofen, naproxen, gemfibrozil and diclofenac, all these four genes were up-regulated by 1.1 ± 0.1- to 15.4 ± 0.6-fold as compared with the control group. For propranolol, increased expression of *traF* and *traP* to 1.7 ± 0.3-fold was detected, but slightly decreased expression of *traB* and *traE* occurred (to 1.1 ± 0.1-fold). Significant increases in pilin gene expression were not detected for iopromide exposure, although we observed decreased expression of *traB*, *traE* and *traF*.

Another contributing factor to conjugation is the direct cell-to-cell contact [48], where fimbriae are important for bacterial cell adhesion. Fimbriae generation and functions are regulated within operons such as *fli*, *fim*, *pil*, *yad*, *ybg* in *E. coli*, and *fli*, *pil*, *flg* in *P. putida* [57–59]. In this study, genes and proteins related to fimbriae adhesion were up-regulated significantly with exposure to the five non-antibiotic pharmaceuticals, except for iopromide (Supplementary Tables S17–S19). For example, in the donor bacterium the gene expression was increased by up to 17.8 ± 0.8-fold under the effect of 0.5 mg/L gemfibrozil (Fig. 4b). While in the recipient bacteria, the highest increase was 4.3 ± 0.3-fold with 0.5 mg/L naproxen (Fig. 4c). In comparison, iopromide exposure repressed expression of most of the fimbriae-related genes in the donor bacteria by 1.2 ± 0.2- to 1.8 ± 0.2-fold.

### Antibiotic-like effects caused by non-antibiotic pharmaceuticals

**Fig. 5:**
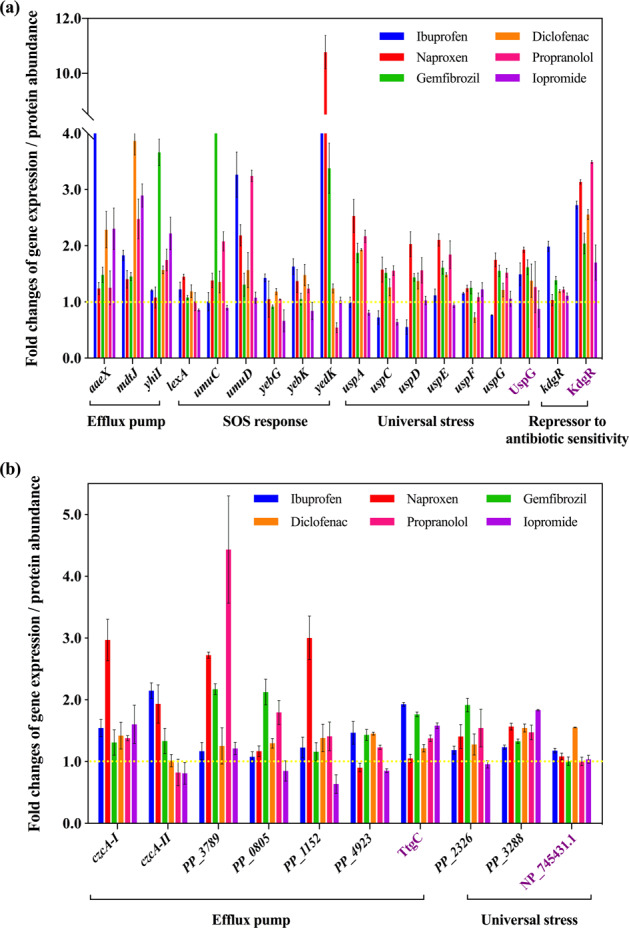
Non-antibiotic pharmaceuticals showed antibiotic-like features on donor (*E. coli* K-12 LE392) and recipient (*P. putida* KT2440) bacteria. **a** Fold changes of expression of core genes and proteins in donor bacteria. **b** Fold changes of expression of core genes and proteins in recipient bacteria. Figures shown are 0.5 mg/L for ibuprofen, naproxen, gemfibrozil, diclofenac, propranolol, and 1.0 mg/L for iopromide. Genes are shown in black, while proteins are shown in purple.

Antibiotics at sub-inhibitory concentrations are known to promote horizontal dissemination of antibiotic resistance, associated with the bacterial SOS response [6, 8]. In this study, we found that non-antibiotic pharmaceuticals also significantly affected the SOS response in both donor and recipient bacteria (Fig. 5, Supplementary Tables S20–S23). Altered gene expression during pharmaceutical exposure was detected for the key regulators of *lexA*, *umu*, *yeb* in the donor, with a total of five genes being affected [60]. With exposure to ibuprofen, naproxen, diclofenac and propranolol, the core genes of the SOS response had up to 4.2 ± 0.3-fold increased expression. Gemfibrozil enhanced the expression of four of the five genes, with the largest change being 5.4 ± 0.4-fold. In contrast, iopromide did not cause any significant expression variation of these five genes. Thus, the SOS response may also contribute to enhanced conjugation with exposure to non-antibiotic pharmaceuticals, and help to explain the differences detected with exposure to different pharmaceuticals.

In addition to the SOS response, the non-antibiotic pharmaceuticals also enhanced expression of efflux pumps, increased levels of universal stress, and elevated levels of repressor genes which regulate antibiotic-sensitivity. Core operons regulating these effects are *mdt*, *usp*, *kdg* in the donor bacterium, and *czc* and *ttg* in the recipient bacterium [61, 62]. Overall, the five non-antibiotic pharmaceuticals caused increased expression of the relevant genes; while exposure to 1 mg/L iopromide had the least effect on gene expression (Supplementary Tables S24–S25).

## Discussion

The spread of antibiotic resistance has posed a serious global threat to public health. Among the dissemination pathways of antibiotic resistance, conjugation, belonging to HGT, possesses significant ecological and evolutionary implications. This is because that conjugation enables the transfer of mobile genetic elements between bacteria, and allows bacterial instantaneous acquisition of multidrug resistance phenotypes from another population [63], and thus exploiting new ecological niches [64].

Pharmaceuticals are being consumed at increasing levels each year, with a 5.8% annual growth in the global pharmaceuticals market, which was worth $935 billion in 2017, and will reach $1170 billion in 2021 [9, 65]. Among the highly-consumed pharmaceuticals, antibiotics comprise only $43 billion or 4.6% of the market. The largest market share comprises non-antibiotic pharmaceuticals [9, 65]. It is well established that antibiotics used at sub-inhibitory concentrations can facilitate the spread of antibiotic resistance [66–70]. However, little is known about the contribution of non-antibiotic, human-targeted pharmaceuticals to the spread of antibiotic resistance.

In this study, we demonstrated that these five commonly consumed non-antibiotic pharmaceuticals (ibuprofen, naproxen, gemfibrozil, diclofenac, and propranolol) can enhance the spread of antibiotic resistance through conjugation at both clinically and environmentally relevant concentrations. Based on two conjugative mating models, this pattern was repeated with two intra- or intergenera populations. This was seen in the enhancement of both absolute transconjugant number and conjugative transfer ratio. The fold changes observed (up to 8-fold) in this study were comparable with- or lower than the conjugation effects caused by sub-inhibitory antibiotics. However, considering human consumption of non-antibiotic pharmaceuticals is wide-spread, the effects caused by these drugs warrant more attention. Moreover, non-antibiotic pharmaceuticals can cause multifaceted effects on the emergence and spread of antibiotic resistance, by increasing expression of efflux pump genes [11], and promoting natural transformation [12], as well as accelerating conjugation, demonstrated in the current study. These findings collectively advance understanding of factors triggering or increasing transfer of mobile genetic elements between bacteria, and highlight the complex and multidimensional nature of the spread of antibiotic resistance in larger bacterial communities.

Additionally, through bacterial culture-, fluorescence-, and advanced molecular methods this study explored the underlying mechanisms related to increased gene transfer [30, 33]. The higher levels of ROS triggered by non-antibiotic pharmaceuticals is a major influence on the increased gene transfer. This was seen from both phenotypic fluorescent ROS detection and genotypic ROS-related gene expression. Moreover, the elevated ROS generation level and enhanced conjugation could be reversed by adding the ROS scavenger. The anaerobic conditions further verified that ROS plays a key role. We previously also reported that carbamazepine facilitates conjugative transfer due to enhanced ROS production [27]. In addition to the non-antibiotic pharmaceuticals, antibiotics, biocides (e.g., triclosan) and heavy metals also increase ROS generation levels, triggering the stress-response in bacteria, thus enhancing the uptake potential of conjugal plasmids [28, 71–74]. Further studies are required to confirm if other non-antibiotic pharmaceuticals follow this pattern of enhancing intracellular ROS generation, thus potentially contributing to increased bacterial gene transfer. Detecting ROS levels in bacteria could potentially screen for non-antibiotic pharmaceuticals that contribute to spreading antibiotic resistance.

In addition to ROS, cell membrane permeability also contributes to enhanced bacterial conjugation. Elevated cell membrane permeability was detected in both the donor and recipient cells with exposure to ibuprofen, naproxen, gemfibrozil, diclofenac, and propranolol, correlating well with the phenotypic conjugative transfer. The outer membrane of Gram-negative bacteria is considered to be a semi-permeable barrier, where increased permeability could enable increased entry of plasmids [63, 75]. Transient membrane permeability also has evolutionary implications, and can facilitate horizontal gene transfer [76]. In this study, it was found that a ROS scavenger could eliminate the effects of the non-antibiotic pharmaceuticals on bacterial cell membrane permeability, indicating that enhanced membrane permeability may have been linked to elevated ROS levels.

Interestingly, it was also found that the non-antibiotic pharmaceuticals caused antibiotic-like bacterial responses, increasing the expression of genes and proteins involved in the SOS response (*lexA*, *umuC*, *umuD* and *soxR*), universal stress (Usp), the efflux pump (*aaeX*, *mdtJ*, *yhiI* and *czcA*), and antibiotic-sensitivity (KdgR). Other in vivo studies show that some pharmaceuticals can cause stress in cells. For example in humans, ibuprofen enhances oxidative stress in plasma during extreme exercise [77], and induces prolonged stress in a rat model [78]. Naproxen can induce oxidative stress and genotoxicity in male Wistar rats [79]. In addition, it has been documented that these non-antibiotic pharmaceuticals can have antimicrobial effects in vivo. NSAIDs have been assessed as potential sources of novel antibacterial agents in vivo [80]. For example, diclofenac protected mice from virulent *Salmonella* infection [81], and ibuprofen demonstrated antimicrobial activity against *Candida albicans* in mouse kidney models [82]. Moreover, it was also reported that human-targeted non-antibiotic drugs cause antibiotic-like side effects on the gut microbiome, and induce antibiotic resistance through activating the efflux pump [11]. The authors determined that bacterial mutant strains lacking TolC, which is responsible for the efflux of antibiotics, became more sensitive to antibiotics and human-targeted non-antibiotic drugs. Further investigations are needed to verify whether these antibiotic-like bacterial responses are directly promoting conjugative transfer.

In this study, we also examined the chemical structures and properties of non-antibiotic pharmaceuticals that may be in common with various antibiotics. Four of the pharmaceuticals, i.e., ibuprofen, naproxen, gemfibrozil, and diclofenac, harbor benzene rings and carboxyl functional groups. This is similar to antibiotics such as ampicillin, cefalexin and ciprofloxacin (Fig. S6). A simple chemical comprising a benzene ring and a carboxyl group is salicylic acid, widely demonstrated to behave like an antibiotic in both Gram-positive and Gram-negative bacteria. This includes reducing bacterial susceptibility towards antimicrobials [83, 84], and inducing intrinsic multiple-antibiotic resistance [85]. In addition, in vitro experiments show that carboxyl functionalized graphene causes structural damage to the plasma membrane, and induces intracellular ROS generation at concentration as low as 4 μg/mL [86]. Carboxyl functionalized graphene also shows toxicity towards *Caenorhabditis elegans*, and enhances ROS production in vivo [87]. Further studies should verify whether the benzene ring or carboxyl group enables certain pharmaceuticals to exhibit antibiotic-like characteristics, and explore other functional groups which may play a role in antibiotic-like effects. In addition, it will also be interesting to further investigate whether and which other pharmaceuticals are able to promote HGT. The relevant research might be facilitated for controlling side effects of these non-antibiotic pharmaceuticals or repurposing of these drugs as antibacterials.

In this study, the in vitro conjugative mating systems were established at laboratory-scale, which cannot reflect the real effects caused by non-antibiotic pharmaceuticals in real environmental or clinical settings. Thus, in vivo animal studies or mixed culture based mating systems should be conducted in the future to evaluate the risk of non-antibiotic human-targeted pharmaceuticals in promoting the dissemination of antibiotic resistance in relevant niches (e.g., human gut or urinary tract systems). For example, we employed pMS6198A (a *bla*_NDM-1_-positive IncA/C plasmid, with resistance to drugs of last resort, including carbapenem), which was originally isolated from a patient suffering from urinary tract infections [15]. Considering these non-antibiotic pharmaceuticals are normally excreted in urine, it will be very relevant to evaluate if these pharmaceuticals can change resistance profiles in urinary tract infections. In addition, given that non-antibiotic pharmaceuticals with higher concentrations decreased the total viable recipient cell number, it would also be useful to analyse the shift of human gut microbiota under long-term exposure of pharmaceuticals. Understanding how a commensal bacterium becomes antibiotic resistant through HGT is one of the central issues in contemporary microbiology and microbial ecology. The expansion of the adaptive potential of microbial populations caused by HGT is still worth investigating.

The findings also urge that it is necessary to assess the possible ecological consequences of the discharge of non-antibiotic pharmaceuticals into the environment, in terms of their antibiotic-like roles. For example, it is worthwhile to explore whether these ubiquitous pharmaceuticals can facilitate the conjugation at a microbial community-wide level in the environment (e.g., in soils or wastewater treatment systems).

## Supplementary information

Supporting Information

## Data Availability

All data was deposited in publicly accessible databases. RNA sequence data are accessible through Gene Expression Omnibus of NCBI (GSE130562).The mass spectrometry proteomics data have been deposited to the ProteomeXchange Consortium via the PRIDE [88] partner repository with the dataset identifier of PXD012642.
