# Supplementary material for: Non-antibiotic pharmaceuticals promote the transmission of multidrug resistance plasmids through intra- and intergenera conjugation
Source: ISME J. 2021 Mar 10;15(9):2493–508. doi: 10.1038/s41396-021-00945-7 (PMC8397710; doi:10.1038/s41396-021-00945-7)
Supplement: Supplementary file 1 — Supporting Information [file 41396_2021_945_MOESM1_ESM.pdf]

Supporting Information

Non-antibiotic pharmaceuticals promote the transmission of multidrug resistance plasmids through intra- and intergenera conjugation

Yue Wang [1], Ji Lu [1], Shuai Zhang [1], Jie Li [1], Likai Mao [1], Zhiguo Yuan [1], Philip L. Bond [1], Jianhua Guo [1]*

[1] Advanced Water Management Centre, The University of Queensland, St. Lucia, Brisbane, Queensland, Australia, 4072

* Corresponding author: j.guo@awmc.uq.edu.au

This file includes:

Supplementary Methods 1 to 7

Supplementary Figures 1 to 6

Supplementary Tables 1 to 25

Supplementary Methods

Text S1. Culture conditions for donor and recipient bacteria

For the environmentally relevant conjugation model (Model-1), both donor and recipient were cultured separately in Luria-Bertani (LB) broth (pH 7.0) at 30 ^{o}C for 16 h with the supplementary of appropriate antibiotics. For donor, 17.0 mg/L tetracycline, 33.0 mg/L kanamycin, and 100.0 mg/L ampicillin were added, while 17.0 mg/L chloramphenicol was dosed in the LB broth for recipient. After culturing, both donor and recipient were washed with phosphate-buffered saline (PBS, pH=7.2) twice to eliminate the possible influence induced by culture media. Afterwards, the donor and recipient were re-suspended separately in different volumes of PBS to obtain initial concentration of 10^8 cfu/ mL based on OD600 values. Then, the donor and recipient were mixed with the ratio of 1:1. The mixtures were applied immediately for the conjugation experiment. Regarding the clinically relevant conjugation model (Model-2), both donor and recipient were cultured separately in LB broth at 37 ^{o}C for 16 h with the supplementary of appropriate antibiotics (100 mg/L ampicillin for donor, and 120 mg/L sodium azide for recipient). Donor and recipient strains were grown to an OD600 value of 1.8. The donor and recipient bacteria were then mixed with a ratio of 1:2 in LB broth for the following conjugation experiment.

Text S2. Determination of MICs

The bacteria with initial concentration of 10^5 cfu/mL were used for MIC detection. In each well of the 96-well plates, 5 μL of the bacteria, 15 μL of antibiotics or non-antibiotic pharmaceuticals with different concentrations, and 130 μL of LB was added. Blank controls were ethanol, sterilized MilliQ water, or dimethyl sulfoxide (DMSO). The 96-well plates were then incubated at 30 ^{o}C for 18 h, followed by OD600 measurement on the plate reader (Tecan Infinite M200, Switzerland). MICs were determined as the concentration of antibiotics or non-antibiotic pharmaceuticals that could inhibit at least 90% of bacterial growth. MICs were tested in triplicate.

Text S3. Selection plates for transconjugant and recipient

The selection plates for environmentally relevant transconjugant in Model-1 contained all of the four kinds of antibiotics (17.0 mg/L tetracycline, 33.0 mg/L kanamycin, and 100.0 mg/L ampicillin), while those for recipient only contained 17.0 mg/L chloramphenicol. After plating on transconjugant and recipient selective plates, the plates were incubated at 30 ^{o}C for 48 h, followed by counting the colonies growing on them. Regarding the clinically relevant

conjugation (i.e., Model-2), selective plates containing 100.0 mg/L ampicillin and 120.0 mg/L sodium azide were applied to count the number of transconjugants, and plates containing 120.0 mg/L sodium azide were used for recipients. After incubation at 37 °C for 24 h, the colonies growing on the selective plates were counted. The results of selection plates are shown as cfu/mL, and transfer ratio was calculated as the number of transconjugants divided by the number of recipients. All the selection plates were performed at least in triplicate. In addition, both donor and recipient were plated onto the transconjugant selective plates, to rule out any spontaneous mutation.

Text S4. PCR conditions

PCR systems were set up as 25 μL, with 12.5 μL Platinum™ Green Hot Start PCR Master Mix (2X) (Invitrogen™), 1 μL 20 μM primer, 1 μL plasmid, and 10.5 μL ddH_2O. Primers are listed in Supplementary table 21. PCR conditions for gene *traF* were: denaturation at 94 °C for 4 min on initial cycle, 30 s for another 35 cycles, annealing at 55 °C for 30 s, extension at 72 °C for 1 min, followed by 7 min. The process was conducted with 30 cycles [1]. PCR conditions for gene *tetA*, bla_{TEM} and bla_{NDM} were the same as those for gene *traF*, except with the annealing temperature of 54 °C, and 50 °C respectively.

Text S5. Sample preparations for transmission electron microscopy

Cells were collected by centrifuging at 4000 g for 5 min, followed by fixing in PBS with 2.5% glutaraldehyde and stored at 4 °C overnight. Afterwards, cell pellets were re-suspended in PBS and microwaved twice at 80 W for 40 s. Following centrifuge, the cell pellets were heated to 37 °C and soaked with 2% agarose. The cell pellets were then solidified, cut into small pieces, added with 1% osmium tetroxide, and microwaved twice at 80 W for 2 min. Osmium tetroxide was then discarded. Samples were further processed by dehydration, infiltration and mounted with Epon and polymerized at 60 °C for 2 days [2]. Ultrathin sections (50–100 nm) of samples were then collected on TEM grid and observed using a 80 kV JEOL JEM-1011 (JEOL, Japan).

Text S6. ROS generation and cell membrane permeability detection

Bacteria strains were washed twice with PBS and resuspended in PBS to 10^6 cfu/mL. For ROS detection, bacteria strains were incubated in dark at 37 °C for 30 min with 2’, 7’-dichlorofluorescein diacetate (DCFDA, at a final concentration of 20 μM, abcam®). Then, 100 μL of the bacteria stained with DCFDA were treated with different concentrations of non-antibiotic pharmaceuticals. 1.5% H_2O_2 was set as positive control, and ethanol or MilliQ

water was set as negative control. After complete mixing by vortex, the mixtures were incubated in dark at 25 °C for 2 h before the detection. During the detection, DCFDA was excited by the 488 nm laser, and DCFDA-stained cells were detected with bandpass filter 525/40 nm.

For cell membrane permeability detection, 100 μL of bacteria strain was exposed to different concentrations of non-antibiotic pharmaceuticals, and incubated at 25 °C for 2 h. The same volume of ethanol or MilliQ water was the negative control, while bacteria strain treated with 100 °C water was the positive control. The strains were then stained with 1 μL of propidium iodide (PI, 2 mM, Life Technologies) and incubated in the dark for 15 min before the cell membrane permeability detection. During the detection, PI was excited by the 561 nm laser, and PI-stained cells were detected with bandpass filter 610/20 nm.

All data was analysed with CytExpert. All the detections were conducted in triplicate. Relative fold increases in ROS production or cell membrane permeability were calculated as pharmaceutical-treated samples divided by corresponding negative control samples according to previous studies [3,4].

Text S7. Proteomics analysis

Qualitative protein libraries were constructed by information dependent analysis (IDA), and quantitative protein determination was based on SWATH-MS. IDA data were combined and searched using ProteinPilot software, with the combined databases of *E. coli* SP only (received from Uniprot on 9th of July 2018) and *P. putida* KT2440 (received from NCBI on 9th of July 2018). Search setting for enzyme digestion was set to trypsin and alkylation was set to iodoacetamide. Afterwards, the constructed IDA library and SWATH-MS data were loaded into PeakView v2.1 for further processing, with the peptide confidence threshold of 99%, number of peptides per protein of 5, and number of transitions per peptide of 3. A minimum of 2 peptides and 3 transitions was used for quantitative analysis.

Supplementary Figures

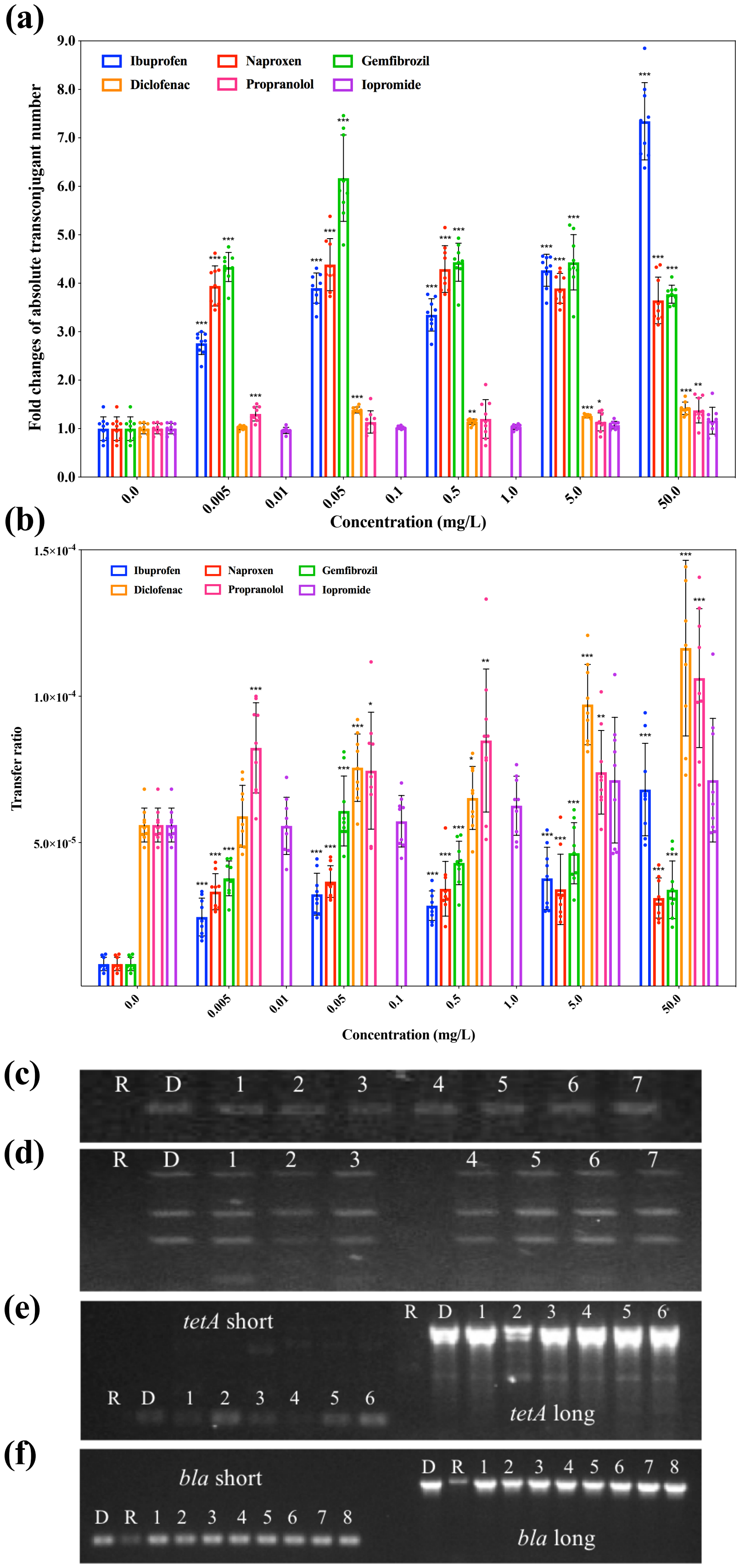

Fig S1. Conjugative ratio of ARGs under the exposure of non-antibiotic pharmaceuticals in environmentally relevant bacterial model (Model-1). (a) Fold change of absolute transconjugant number. (b) Transfer ratio. (c) Electrophoresis of RP4 plasmid (lanes R, D, and 1-7, refer to plasmids extracted from recipient, donor, and transconjugants of different pharmaceutical-dosed groups). (d) Electrophoresis of RP4 plasmid detection using specific primers (lanes R, D, and 1-7, refer to plasmids extracted from recipient, donor, and transconjugants of different pharmaceutical-dosed groups). (e) Electrophoresis of plasmid PCR products for *tetA* gene (lanes R, D, and 1-6, refer to plasmids extracted from recipient, donor, and transconjugants of different pharmaceutical-dosed groups). (f) Electrophoresis of plasmid PCR products for *bla* gene (lanes R, D, and 1-8, refer to plasmids extracted from recipient, donor, and transconjugants of different pharmaceutical-dosed groups). Significant differences between non-antibiotic-dosed samples and the control were analyzed by independent-sample *t* test, and corrected by Benjamini–Hochberg method for multiple comparisons, *P<0.05, **P<0.01, and ***P<0.001.

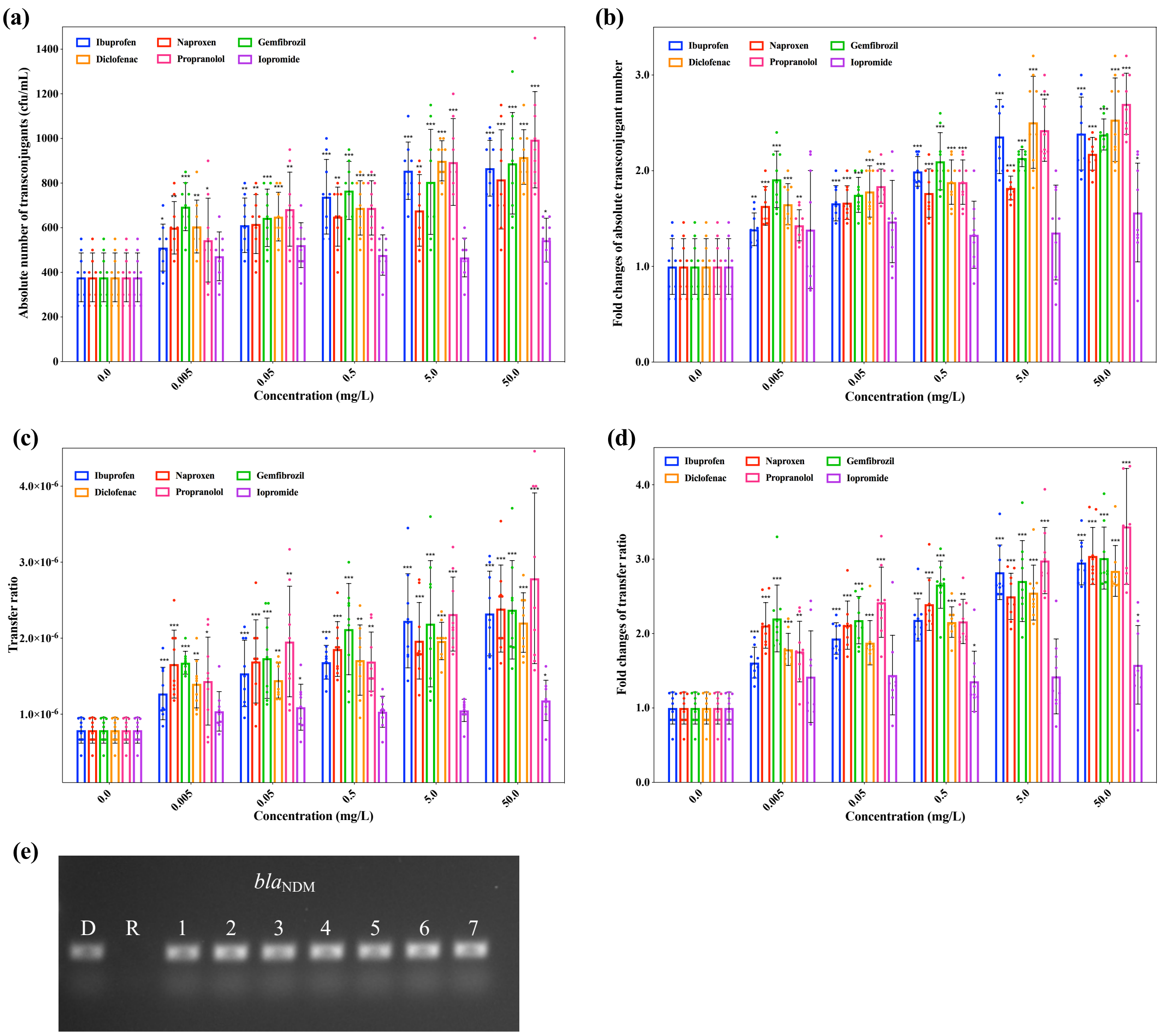

Fig S2. Effects of non-antibiotic pharmaceuticals on the conjugative transfer of ARGs in clinically relevant bacterial model (Model-2). (a) Absolute number of transconjugants under the exposure of non-antibiotic pharmaceuticals. (b) Fold changes of transconjugants' absolute number under the exposure of non-antibiotic pharmaceuticals. (c) Transfer ratio under the exposure of non-antibiotic pharmaceuticals. (d) Fold changes of transfer ratio under the exposure of non-antibiotic pharmaceuticals. (e) Electrophoresis of plasmid PCR products for *bla*$_{NDM}$ gene (lanes D, R, and 1-7, refer to plasmids extracted from donor, recipient, and transconjugants of different pharmaceutical-dosed groups). Significant differences between non-antibiotic-dosed samples and the control were analyzed by independent-sample *t* test, and corrected by Benjamini–Hochberg method for multiple comparisons, *$P<0.05$, **$P<0.01$, and ***$P<0.001$.

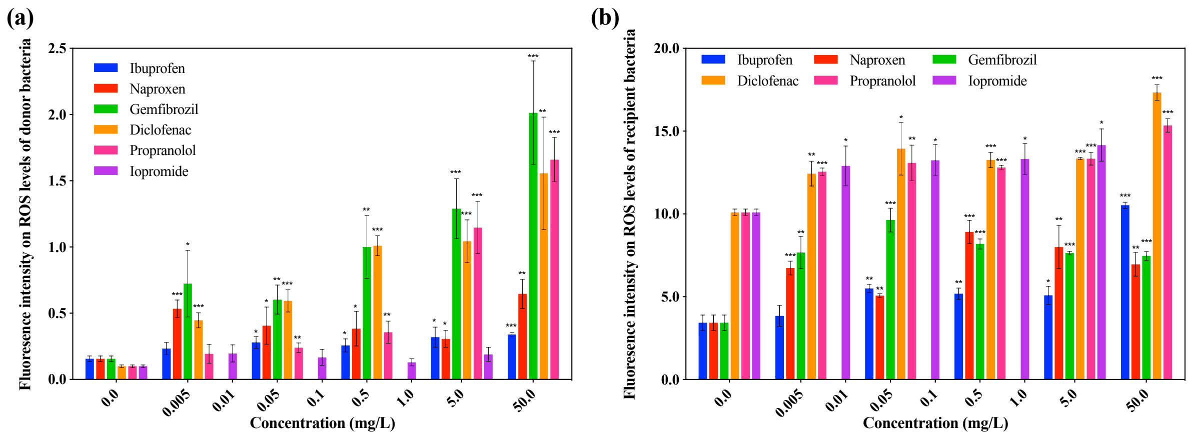

Fig S3. Effects of non-antibiotic pharmaceuticals on ROS in the donor (*E. coli* K-12 LE392) and recipient (*P. putida* KT2440) bacteria in Model-1. (a) Fluorescence intensity on ROS levels of donor bacteria. (b) Fluorescence intensity on ROS levels of recipient bacteria. Significant differences between non-antibiotic-dosed samples and the control were analyzed by independent-sample *t* test, and corrected by Benjamini–Hochberg method for multiple comparisons, *P<0.05, **P<0.01, and ***P<0.001.

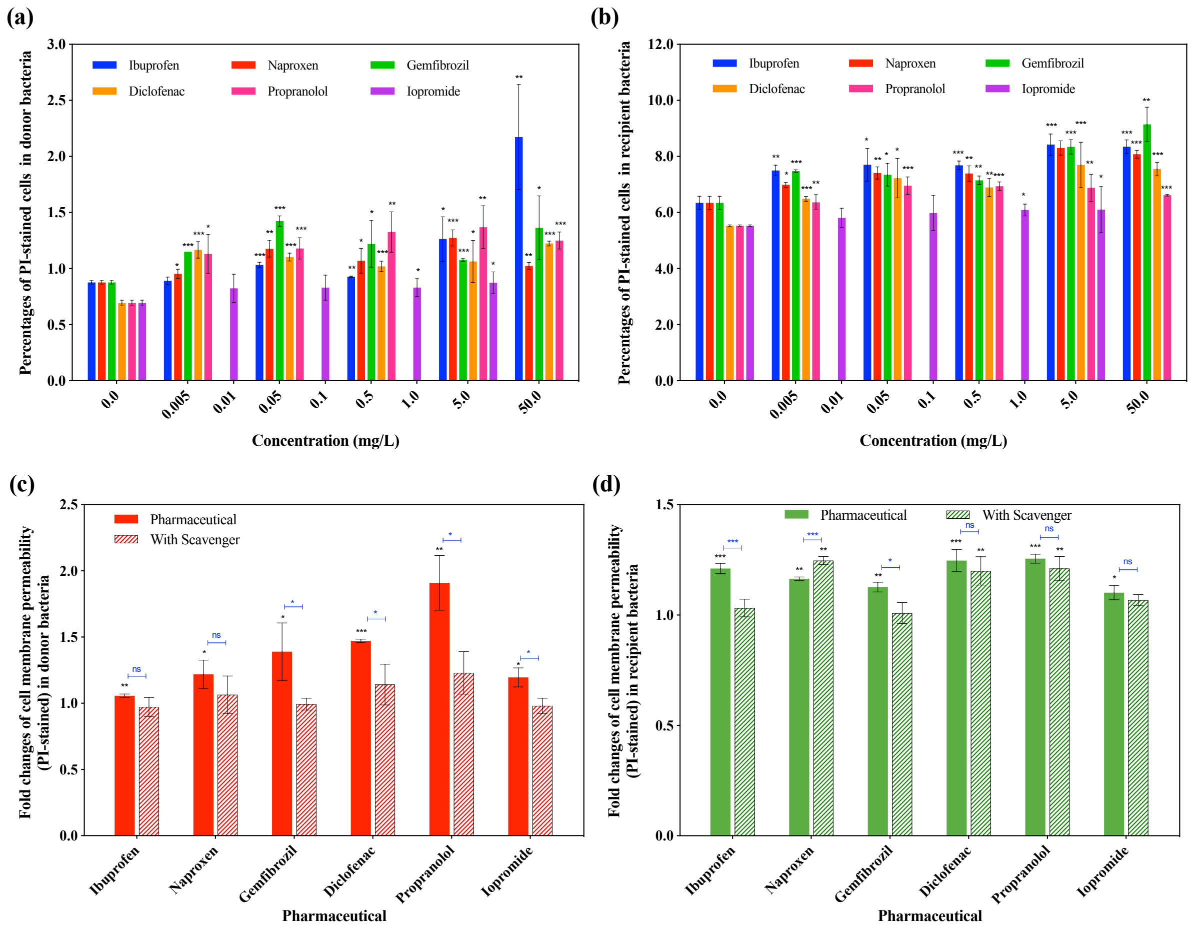

Fig S4. Effects of non-antibiotic pharmaceuticals on cell membranes in the donor (*E. coli* K-12 LE392) and recipient (*P. putida* KT2440) bacteria in Model-1. (a) Percentages of PI-stained cells in donor bacteria. (b) Percentages of PI-stained cells in recipient bacteria. (c) Fold change of cell membrane permeability with/without ROS scavenger in donor bacteria. (d) Fold change of cell membrane permeability with/without ROS scavenger in recipient bacteria. Significant differences between non-antibiotic-dosed samples and the control were analyzed by independent-sample *t* test, **P*<0.05, ***P*<0.01, and ****P*<0.001. For (c)-(d), figures shown are 0.5 mg/L for ibuprofen, naproxen, gemfibrozil, diclofenac, propranolol, and 1.0 mg/L for iopromide.

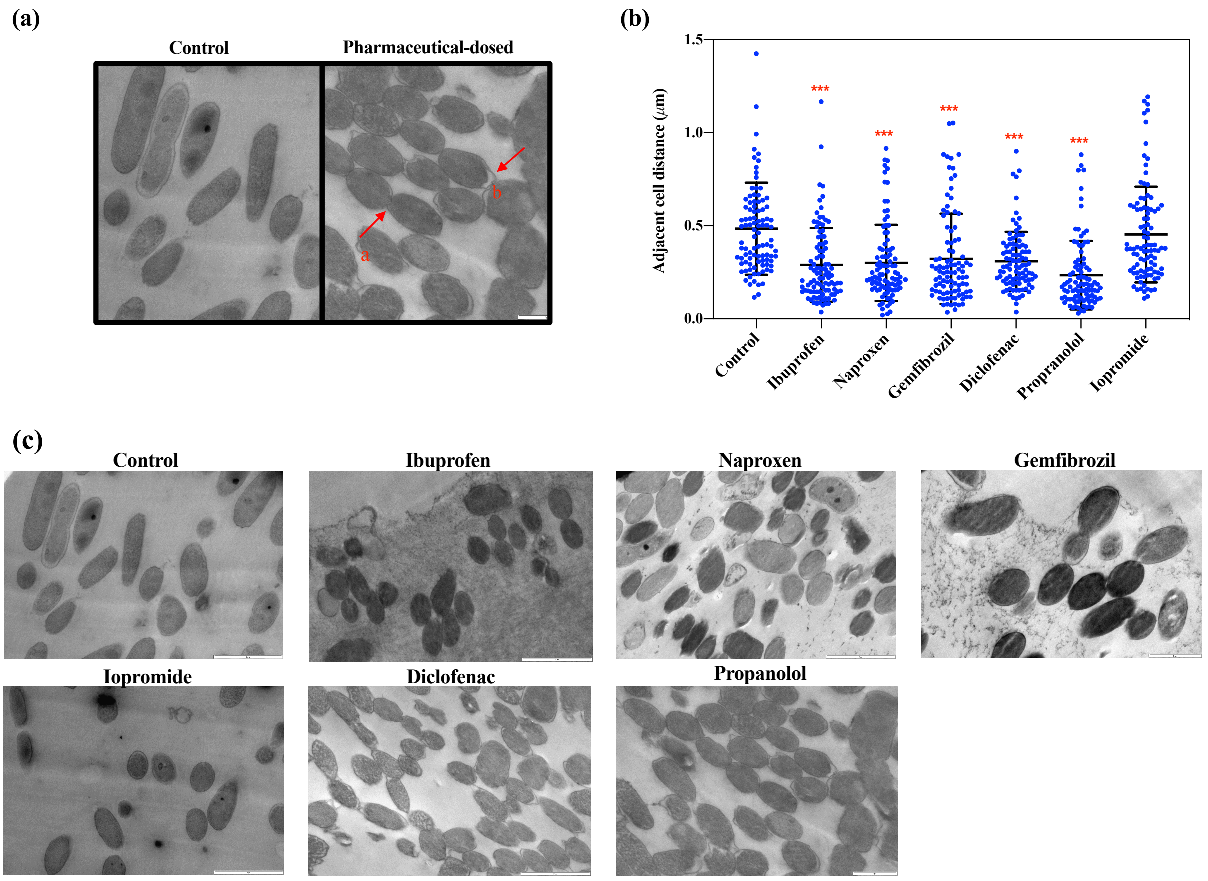

Fig S5. Transmission electron microscopy (TEM) images of donor and recipient bacteria under the exposure of pharmaceuticals in Model-1. (a) Cells remained separate and intact in the control group; while cells became closer (arrow a) and membranes were partially damaged (arrow b) with pharmaceutical dosage. (b) Adjacent cell distance under the exposure of non-antibiotic pharmaceuticals (n=100). (c) Images of donor and recipient bacteria under the exposure of non-antibiotic pharmaceuticals.

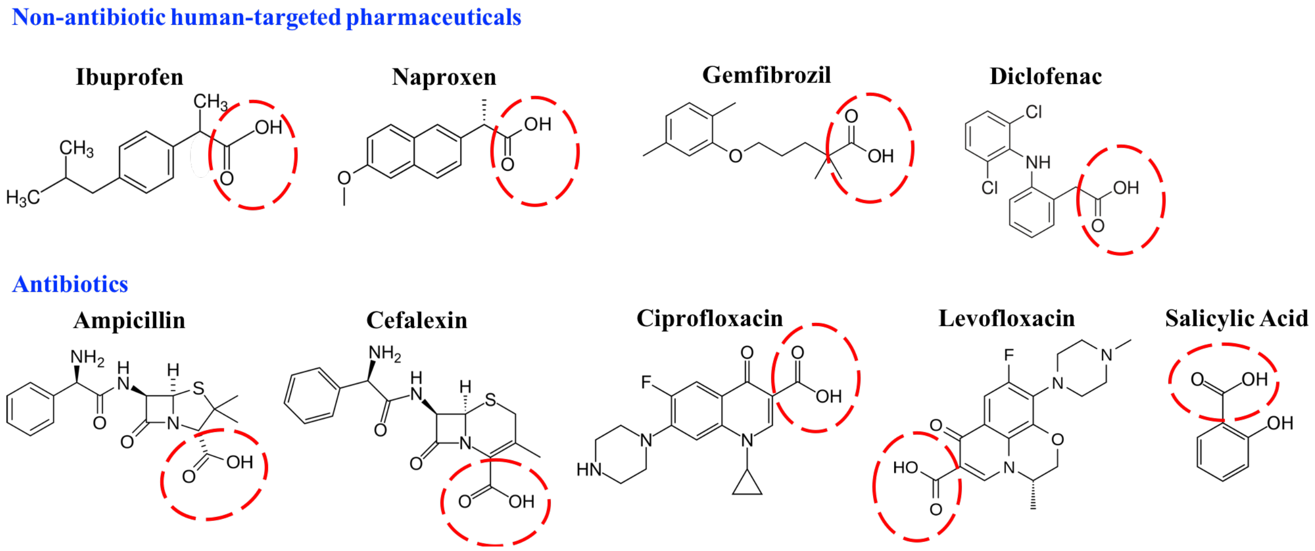

Fig S6. Chemical structures of non-antibiotic human-targeted pharmaceuticals that can promote conjugation, and chemical structures of commonly prescribed antibiotics. All of these drugs harbour benzene ring and carboxyl functional group.

Supplementary Tables

Table S1. Primers used in this study [1,5]

Gene	Primer	Sequence of primer
tetA	Short FW	GACTATCGTCGCCGCACTTA
	Short RV	ATAATGGCCTGCTTCTCGCC
	Long FW	CGTGTATGAAATCTAACAATGCGCT
	Long RV	CCATTCAGGTCGAGGTGGC
bla$_{TEM}$	Short FW	AATAAACCAGCCAGCCGGAA
	Short RV	TTGATCGTTGGGAACCGGAG
	Long FW	TTACCAATGCTTAATCAGTGAGGC
	Long RV	ATGAGTATTCAACATTTCCGTGTCG
traF	FW	AAGTGTTCAGGGTGCTTCTGC
	RV	GTCGCCTTAACCGTGGTGTT
bla$_{NDM}$	FW	CGATCTGGTTTTCCGCCAGC
	RV	GGTCTGGTCATCGGTCCAGG

Table S2. Minimum inhibitory concentrations (MICs) of donor and recipient bacterial strains towards non-antibiotic pharmaceuticals

Strains	MICs (mg/L)					
	Ibuprofen	Naproxen	Gemfibrozil	Iopromide	Diclofenac	Propranolol
Donor (*E. coli* LE392)	500	500	>500	>10	>500	500
Recipient (*P. putida* KT2440)	500	500	>500	>50	>1000	500
Donor (*E. coli* MG1655)	500	500	>500	>50	>500	500
Recipient (*E. coli* J53)	500	500	500	>50	>500	500

Table S3. Results of conjugative transfer under the exposure of low concentrations of non-antibiotic pharmaceuticals dissolved in MilliQ water (From *E. coli* harboring RP4 plasmid to *P. putida*)[#]

Condition	Absolute number of transconjugant (cfu/mL)	Fold change of transconjugant absolute number	Transfer ratio (number of transconjugant / number of recipient)	Fold change of transfer ratio
Control (MilliQ water)	2675.56±280.28	1.00±0.10	$4.15\times10^{-5}\pm3.58\times10^{-6}$	1.00±0.09
Ibuprofen 0.005 mg/L	4148.89±308.56	1.56±0.07	$6.87\times10^{-5}\pm5.72\times10^{-6}$	1.66±0.04
Ibuprofen 0.05 mg/L	4395.56±433.67	1.64±0.03	$7.89\times10^{-5}\pm1.01\times10^{-5}$	1.90±0.12
Naproxen 0.005 mg/L	4637.78±416.91	1.74±0.05	$8.08\times10^{-5}\pm1.04\times10^{-5}$	1.94±0.10
Naproxen 0.05 mg/L	4706.67±496.74	1.76±0.05	$8.44\times10^{-5}\pm1.11\times10^{-5}$	2.03±0.12
Gemfibrozil 0.005 mg/L	5048.89±282.23	1.90±0.10	$8.75\times10^{-5}\pm8.81\times10^{-6}$	2.11±0.05
Gemfibrozil 0.05 mg/L	4797.78±676.36	1.79±0.08	$8.38\times10^{-5}\pm1.36\times10^{-5}$	2.01±0.17

[#] Results are shown as mean ± SD, n=9; Significant differences between non-antibiotic dosed samples and the control were analyzed by independent-sample *t* test and corrected by Benjamini–Hochberg method for multiple comparisons, all of the calculated *P* values were less than 0.001.

Table S4. Total viable recipient number and corresponding transfer ratio under the exposure of non-antibiotic pharmaceuticals (environmentally relevant conjugation, Model-1, From *E. coli* harboring RP4 plasmid to *P. putida*)[*]

	Concentration (mg/L)	Ibuprofen	Naproxen	Gemfibrozil	Diclofenac	Propranolol	Iopromide [#]
Total viable recipient (cfu/mL)	0	$4.7\times10^7\pm5.5\times10^6$	$4.7\times10^7\pm5.5\times10^6$	$4.7\times10^7\pm5.5\times10^6$	$7.6\times10^7\pm6.0\times10^6$	$7.6\times10^7\pm6.0\times10^6$	$7.6\times10^7\pm6.0\times10^6$
	0.005	$4.5\times10^7\pm5.2\times10^6$	$4.7\times10^7\pm4.7\times10^6$	$4.6\times10^7\pm6.8\times10^6$	$7.5\times10^7\pm1.0\times10^7$	$6.8\times10^7\pm1.2\times10^6$	$7.4\times10^7\pm6.4\times10^6$
	0.05	$4.8\times10^7\pm6.2\times10^6$	$4.7\times10^7\pm5.2\times10^6$	$4.0\times10^7\pm4.1\times10^6$	$7.8\times10^7\pm7.5\times10^6$	$6.6\times10^7\pm1.2\times10^7$	$7.6\times10^7\pm6.5\times10^6$
	0.5	$4.7\times10^7\pm6.9\times10^6$	$5.1\times10^7\pm7.3\times10^6$	$4.1\times10^7\pm3.5\times10^6$	$7.5\times10^7\pm4.0\times10^6$	$5.9\times10^7\pm6.2\times10^6$	$7.1\times10^7\pm8.5\times10^6$
	5.0	$4.6\times10^7\pm6.8\times10^6$	$4.7\times10^7\pm9.1\times10^6$	$3.8\times10^7\pm5.8\times10^6$	$5.6\times10^7\pm9.6\times10^6$	$6.5\times10^7\pm5.8\times10^6$	$6.7\times10^7\pm1.1\times10^7$
	50.0	$4.3\times10^7\pm4.3\times10^6$	$4.7\times10^7\pm6.8\times10^6$	$4.5\times10^7\pm5.9\times10^6$	$5.4\times10^7\pm1.1\times10^7$	$5.5\times10^7\pm7.2\times10^6$	$7.0\times10^7\pm9.9\times10^6$
Fold change of total viable recipient number	0.005	0.96±0.12 (ns)	1.00±0.13 (ns)	0.97±0.16 (ns)	1.00±0.21 (ns)	0.90±0.14 (ns)	0.99±0.15 (ns)
	0.05	1.03±0.16 (ns)	1.01±0.21 (ns)	0.85±0.13 (ns)	1.04±0.18 (ns)	0.89±0.24 (ns)	1.01±0.08 (ns)
	0.5	1.00±0.18 (ns)	1.09±0.27 (ns)	0.88±0.15 (*)	0.99±0.09 (ns)	0.78±0.08 (***)	0.94±0.07 (ns)
	5.0	0.98±0.16 (ns)	1.01±0.18 (ns)	0.82±0.18 (**)	0.74±0.12 (***)	0.87±0.13 (*)	0.89±0.14 (ns)
	50.0	0.92±0.13 (ns)	1.00±0.16 (ns)	0.98±0.20 (ns)	0.72±0.16 (***)	0.73±0.06 (***)	0.93±0.13 (ns)
Fold change of transfer ratio	0.005	2.89±0.24 (***)	3.98±0.49 (***)	4.54±0.52 (***)	1.05±0.09 (ns)	1.46±0.16 (***)	0.96±0.06 (ns)
	0.05	3.84±0.33 (***)	4.42±0.57 (***)	7.25±0.69 (***)	1.34±0.09 (***)	1.31±0.21 (**)	1.02±0.03 (ns)
	0.5	3.41±0.35 (***)	4.03±0.37 (***)	5.15±0.53 (***)	1.16±0.09 (**)	1.49±0.27 (***)	1.04±0.05 (ns)
	5.0	4.43±0.48 (***)	3.95±0.50 (***)	5.48±0.36 (***)	1.73±0.09 (***)	1.31±0.11 (***)	1.07±0.07 (ns)
	50.0	8.06±0.59 (***)	3.69±0.26 (***)	3.96±0.26 (***)	2.05±0.33 (***)	1.88±0.25 (***)	1.16±0.26 (ns)

[*] n=9, data are shown as mean ± SD, fold changes were in comparison with the corresponding control values. Significant differences between non-antibiotic dosed samples and the control were analyzed by independent-sample *t* test and corrected by Benjamini–Hochberg method for multiple comparisons, ns $P > 0.05$, $*P < 0.05$, $**P < 0.01$, and $***P < 0.001$.

[#] The concentrations for iopromide are 0.01, 0.1, 1, 5, 50 mg/L, respectively

Table S5. Minimum inhibitory concentrations (MICs) of donor, recipient, and different transconjugants towards antibiotics*

Antibiotics	MICs (mg/L)									
	Donor	Recipient	TC 1	TC 2	TC 3	TC 4	TC 5	TC 6	TC 7	TC 8
Tetracycline	12.5	1.25	12.5	12.5	12.5	12.5	12.5	12.5	12.5	12.5
Kanamycin	20	5	20	20	20	20	20	20	20	20
Ampicillin	>120	6	>120	>120	>120	>120	>120	>120	>120	>120
Chloram-phenicol	0.13	0.39	0.39	0.39	0.39	0.39	0.39	0.39	0.39	0.39

* TC 1-8: transconjugants in mating system treated with Milli-Q water, ethanol, ibuprofen, naproxen, gemfibrozil, iopromide, diclofenac, Propranolol, respectively

Table S6. The calculated maximum growth rates of recipient and transconjugant under the exposure of non-antibiotic pharmaceuticals [#]

Unit: h^{-1}

	Concentration (mg/L)	Ibuprofen	Naproxen	Gemfibrozil	Diclofenac	Propranolol	Iopromide [#]
Recipient (*P. putida* KT2440)	0	0.844±0.015	0.844±0.015	0.844±0.015	0.853±0.015	0.853±0.015	0.853±0.015
	0.005	0.857±0.011<br>(*P*=0.296)	0.861±0.013<br>(*P*=0.217)	0.831±0.001<br>(*P*=0.217)	0.862±0.009<br>(*P*=0.436)	0.866±0.027<br>(*P*=0.507)	0.875±0.004<br>(*P*=0.076)
	0.05	0.851±0.017<br>(*P*=0.633)	0.857±0.018<br>(*P*=0.399)	0.831±0.001<br>(*P*=0.229)	0.864±0.005<br>(*P*=0.310)	0.883±0.010<br>(*P*=0.055)	0.861±0.001<br>(*P*=0.439)
	0.5	0.829±0.027<br>(*P*=0.449)	0.834±0.005<br>(*P*=0.343)	0.851±0.016<br>(*P*=0.595)	0.853±0.001<br>(*P*=0.994)	0.842±0.017<br>(*P*=0.433)	0.866±0.008<br>(*P*=0.254)
	5.0	0.830±0.008<br>(*P*=0.242)	0.842±0.018<br>(*P*=0.883)	0.872±0.010<br>(*P*=0.052)	0.858±0.005<br>(*P*=0.625)	0.857±0.004<br>(*P*=0.701)	0.858±0.025<br>(*P*=0.787)
	50.0	0.839±0.010<br>(*P*=0.667)	0.832±0.020<br>(*P*=0.475)	0.838±0.022<br>(*P*=0.716)	0.845±0.016<br>(*P*=0.535)	0.865±0.013<br>(*P*=0.356)	0.856±0.030<br>(*P*=0.916)
Transconjugant (*P. putida* with RP4 plasmid)	0	0.827±0.030	0.827±0.030	0.827±0.030	0.824±0.022	0.824±0.022	0.824±0.022
	0.005	0.833±0.029<br>(*P*=0.838)	0.829±0.027<br>(*P*=0.957)	0.818±0.006<br>(*P*=0.615)	0.837±0.020<br>(*P*=0.487)	0.823±0.013<br>(*P*=0.966)	0.840±0.014<br>(*P*=0.349)
	0.05	0.834±0.018<br>(*P*=0.760)	0.831±0.005<br>(*P*=0.857)	0.829±0.008<br>(*P*=0.942)	0.829±0.026<br>(*P*=0.804)	0.811±0.002<br>(*P*=0.369)	0.814±0.015<br>(*P*=0.565)
	0.5	0.831±0.037<br>(*P*=0.888)	0.823±0.015<br>(*P*=0.826)	0.831±0.004<br>(*P*=0.851)	0.838±0.014<br>(*P*=0.420)	0.821±0.001<br>(*P*=0.860)	0.836±0.019<br>(*P*=0.501)
	5.0	0.830±0.006<br>(*P*=0.874)	0.828±0.030<br>(*P*=0.986)	0.834±0.007<br>(*P*=0.727)	0.828±0.021<br>(*P*=0.810)	0.827±0.024<br>(*P*=0.863)	0.822±0.068<br>(*P*=0.966)
	50.0	0.829±0.005<br>(*P*=0.913)	0.815±0.006<br>(*P*=0.521)	0.838±0.019<br>(*P*=0.631)	0.811±0.013<br>(*P*=0.422)	0.837±0.029<br>(*P*=0.560)	0.828±0.005<br>(*P*=0.771)

The calculation was based on Gompertz Model, and Curve Fitting Tool in Matlab R2015b was applied. Data were shown as mean±SD. Significant differences between non-antibiotic-dosed groups and the corresponding control group were calculated using independent-sample *t* test, and corrected by Benjamini–Hochberg method for multiple comparisons. Concentrations for iopromide were 0.01, 0.1, 1.0, 5.0, 50.0 mg/L, respectively.

Table S7. Total viable recipient number and corresponding transfer ratio under the exposure of non-antibiotic pharmaceuticals (clinically relevant conjugation, Model-2, From *E. coli* harboring pMS6198A plasmid to *E. coli*)*

	Concentration (mg/L)	Ibuprofen	Naproxen	Gemfibrozil	Diclofenac	Propranolol	Iopromide #
Total viable recipient (cfu/mL)	0	$4.8\times10^8\pm7.5\times10^7$	$4.8\times10^8\pm7.5\times10^7$	$4.8\times10^8\pm7.5\times10^7$	$4.8\times10^8\pm7.5\times10^7$	$4.8\times10^8\pm7.5\times10^7$	$4.8\times10^8\pm7.5\times10^7$
	0.005	$4.1\times10^8\pm5.8\times10^7$	$3.7\times10^8\pm6.6\times10^7$	$4.1\times10^8\pm5.2\times10^7$	$4.4\times10^8\pm7.3\times10^7$	$4.0\times10^8\pm6.6\times10^7$	$4.6\times10^8\pm5.7\times10^7$
	0.05	$4.1\times10^8\pm6.8\times10^7$	$3.8\times10^8\pm7.0\times10^7$	$3.8\times10^8\pm5.1\times10^7$	$4.5\times10^8\pm4.2\times10^7$	$3.7\times10^8\pm7.1\times10^7$	$4.9\times10^8\pm5.0\times10^7$
	0.5	$4.4\times10^8\pm7.8\times10^7$	$3.5\times10^8\pm5.6\times10^7$	$3.8\times10^8\pm7.9\times10^7$	$4.2\times10^8\pm7.2\times10^7$	$4.1\times10^8\pm5.5\times10^7$	$4.7\times10^8\pm5.7\times10^7$
	5.0	$4.0\times10^8\pm7.6\times10^7$	$3.5\times10^8\pm7.3\times10^7$	$3.9\times10^8\pm7.6\times10^7$	$4.6\times10^8\pm6.2\times10^7$	$3.9\times10^8\pm8.9\times10^7$	$4.4\times10^8\pm5.2\times10^7$
	50.0	$3.9\times10^8\pm7.2\times10^7$	$3.4\times10^8\pm6.4\times10^7$	$3.8\times10^8\pm4.9\times10^7$	$4.2\times10^8\pm5.8\times10^7$	$3.9\times10^8\pm8.4\times10^7$	$4.7\times10^8\pm3.7\times10^7$
Fold change of total viable recipient number	0.005	0.89±0.21 (ns)	0.79±0.15 (ns)	0.87±0.08 (ns)	0.94±0.17 (ns)	0.85±0.17 (ns)	0.99±0.22 (ns)
	0.05	0.89±0.24 (ns)	0.83±0.24 (ns)	0.81±0.12 (ns)	0.97±0.19 (ns)	0.79±0.19 (ns)	1.05±0.21 (ns)
	0.5	0.95±0.25 (ns)	0.75±0.13 (*)	0.83±0.28 (ns)	0.91±0.26 (ns)	0.88±0.15 (ns)	0.99±0.14 (ns)
	5.0	0.86±0.23 (ns)	0.78±0.27 (ns)	0.82±0.16 (ns)	1.00±0.23 (ns)	0.84±0.22 (ns)	0.94±0.12 (ns)
	50.0	0.82±0.17 (ns)	0.74±0.17 (*)	0.81±0.17 (ns)	0.90±0.19 (ns)	0.84±0.25 (ns)	1.00±0.18 (ns)
Fold change of transfer ratio	0.005	1.61±0.20 (***)	2.11±0.29 (***)	2.20±0.43 (***)	1.79±0.21 (***)	1.76±0.39 (***)	1.42±0.58 (ns)
	0.05	1.93±0.20 (***)	2.11±0.31 (***)	2.18±0.30 (***)	1.88±0.28 (***)	2.42±0.45 (***)	1.44±0.50 (ns)
	0.5	2.18±0.27 (***)	2.39±0.33 (***)	2.66±0.30 (***)	2.15±0.19 (***)	2.16±0.28 (***)	1.36±0.38 (ns)
	5.0	2.82±0.35 (***)	2.50±0.29 (***)	2.71±0.51 (***)	2.55±0.35 (***)	2.98±0.42 (***)	1.42±0.48 (ns)
	50.0	2.95±0.28 (***)	3.04±0.36 (***)	3.02±0.39 (***)	2.84±0.32 (***)	3.44±0.74 (***)	1.58±0.50 (*)

* n=9, data are shown as mean ± SD, fold changes were in comparison with the corresponding control values. Significant differences between non-antibiotic dosed samples and the control were analyzed by independent-sample *t* test and corrected by Benjamini–Hochberg method for multiple comparisons, ns $P > 0.05$, *$P < 0.05$, **$P < 0.01$, and ***$P < 0.001$.

The concentrations for iopromide are 0.01, 0.1, 1, 5, 50 mg/L, respectively

Table S8. Genes relevant to ROS production in donor bacteria *E. coli* K-12 LE392 after exposure of non-antibiotic pharmaceuticals

**Gene**	**COG Annotation**	**Fold Change of FPKM** *					
		**Ibuprofen**	**Naproxen**	**Gemfibrozil**	**Diclofenac**	**Propranolol**	**Iopromide**
ahpC	Alkyl hydroperoxide reductase subunit AhpC (peroxiredoxin)	1.44	1.33	1.04	1.39	1.37	1.32
ahpF	Alkyl hydroperoxide reductase subunit AhpF	1.07	0.94	0.85	1.06	1.04	1.13
alkB	Alkylated DNA repair dioxygenase AlkB	1.19	4.79	1.69	1.60	2.14	1.56
oxyR	DNA-binding transcriptional regulator, LysR family	1.51	1.31	1.04	1.27	1.15	1.06
rutC	Enamine deaminase RidA, house cleaning of reactive enamine intermediates, YjgF/YER057c/UK114 family	1.34	1.48	10.85	1.64	1.35	1.11
rutE	Homoserine acetyltransferase	2.23	10.56	1.29	1.65	1.26	1.31
sodB	Superoxide dismutase	1.55	1.40	1.03	1.03	1.20	1.19
sodC	Superoxide dismutase	1.46	2.27	1.01	1.01	1.22	1.01

Gene	COG Annotation	**Fold Change of FPKM** *					
		**Ibuprofen**	**Naproxen**	**Gemfibrozil**	**Diclofenac**	**Propranolol**	**Iopromide**
soxR	DNA-binding transcriptional regulator, MerR family	1.49	1.08	1.09	1.10	1.57	1.16
soxS	AraC-type DNA-binding domain and AraC-containing proteins	1.25	1.16	1.04	1.27	0.95	1.03
trxC	Negative regulator of GroEL, contains thioredoxin-like and TPR-like domains	2.77	2.06	1.25	2.00	2.81	0.94

*: Comparing with the control group without pharmaceutical dosage

Table S9. Proteins relevant to ROS production in donor bacteria *E. coli* K-12 LE392 after exposure of non-antibiotic pharmaceuticals

**Protein**	**Description**	**Fold Change of Protein Abundance** *					
		**Ibuprofen**	**Naproxen**	**Gemfibrozil**	**Diclofenac**	**Propranolol**	**Iopromide**
AhpF	Alkyl hydroperoxide reductase subunit F	0.93	1.02	1.06	1.08	1.12	1.05
SodC	Superoxide dismutase	2.17	2.25	1.46	2.30	4.72	2.27

*: Comparing with the control group without pharmaceutical dosage

Table S10. Genes relevant to ROS production in recipient bacteria *P. putida* KT2440 after exposure of non-antibiotic pharmaceuticals

Gene	COG Annotation	Fold Change of FPKM *					
		**Ibuprofen**	**Naproxen**	**Gemfibrozil**	**Diclofenac**	**Propranolol**	**Iopromide**
oxyR	Oxidative and nitrosative stress transcriptional dual regulator	1.54	1.27	1.17	1.14	1.11	1.04
sodA	Superoxide dismutase	1.40	2.07	3.32	1.37	2.66	1.34
sodB	Superoxide dismutase	1.17	1.21	1.02	1.11	1.05	0.95
soxD	Sarcosine oxidase subunit delta	1.60	2.55	1.24	0.52	1.64	1.55
soxR	DNA-binding transcriptional regulator	2.00	1.69	1.69	3.94	4.17	4.08

*: Comparing with the control group without pharmaceutical dosage

Table S11. Proteins relevant to ROS production in recipient bacteria *P. putida* KT2440 after exposure of non-antibiotic pharmaceuticals

**Protein**	**Description**	**Fold Change of Protein Abundance** *					
		**Ibuprofen**	**Naproxen**	**Gemfibrozil**	**Diclofenac**	**Propranolol**	**Iopromide**
AhpC	Alkyl hydroperoxide reductase subunit C	2.73	1.73	1.87	1.31	1.08	1.95
SodF	Superoxide dismutase	1.07	0.91	0.99	0.95	0.62	0.76
Tpx	Thioredoxin peroxidase	1.40	1.08	1.12	0.90	0.95	1.21

*: Comparing with the control group without pharmaceutical dosage

Table S12. Genes relevant to cell membrane in donor bacteria *E. coli* K-12 LE392 after exposure of non-antibiotic pharmaceuticals

Gene	COG Annotation	Fold Change of FPKM *					
		Ibuprofen	Naproxen	Gemfibrozil	Diclofenac	Propranolol	Iopromide
csgG	Curli production assembly/transport outer membrane lipoprotein	3.73	4.72	1.54	3.05	1.77	1.13
cusA	Efflux system membrane component	1.25	1.26	2.07	0.75	1.01	0.67
ompC	Outer membrane porin protein C	1.19	1.08	1.24	1.07	1.12	0.94
ompF	Outer membrane porin 1a	2.51	2.38	1.02	1.82	2.00	1.04
ompN	Outer membrane pore protein non-specific	1.11	1.32	1.39	2.35	1.69	1.04
ompR	Response regulator in two-component regulatory system	1.03	1.23	1.01	1.37	1.35	1.51
ompT	Outer membrane protease	1.45	1.21	0.35	1.21	1.26	0.61
ompW	Outer membrane protein W	0.70	1.12	1.74	1.92	1.18	0.86

Gene	COG Annotation	Fold Change of FPKM *					
		Ibuprofen	Naproxen	Gemfibrozil	Diclofenac	Propranolol	Iopromide
pgaA	Biofilm adhesin polysaccharide PGA secretin OM porin export protein	1.80	2.55	2.93	2.75	2.25	1.27
ybhG	Putative membrane fusion protein (MFP) component of efflux pump membrane anchor	1.16	1.14	4.72	1.80	2.33	2.97
ydcU	Putative ABC transporter permease	7.26	4.20	2.36	1.61	7.94	1.09
yfaZ	Outer membrane protein putative porin	1.53	1.78	1.31	1.31	1.51	1.11

*: Comparing with the control group without pharmaceutical dosage

Table S13. Proteins relevant to cell membrane in donor bacteria *E. coli* K-12 LE392 after exposure of non-antibiotic pharmaceuticals

Protein	Description	Fold Change of Protein Abundance *					
		Ibuprofen	Naproxen	Gemfibrozil	Diclofenac	Propranolol	Iopromide
BamB	Outer membrane protein assembly factor	0.77	0.93	0.96	1.06	1.21	1.19
OmpC	Outer membrane protein C	1.28	1.54	1.59	1.51	2.16	2.06
OmpF	Outer membrane protein F	1.61	1.52	1.20	1.57	1.62	2.30
Slp	Outer membrane protein	0.72	0.84	0.83	1.06	0.99	0.81

*: Comparing with the control group without pharmaceutical dosage

Table S14. Genes relevant to cell membrane in recipient bacteria *P. putida* KT2440 after exposure of non-antibiotic pharmaceuticals

Gene	COG Annotation	Fold Change of FPKM *					
		**Ibuprofen**	**Naproxen**	**Gemfibrozil**	**Diclofenac**	**Propranolol**	**Iopromide**
czcB-I	Function of homologous gene experimentally demonstrated in another organism Product type m: membrane componentTransport and binding proteins	1.49	3.86	1.93	1.33	1.06	1.83
czcC	RND transporter outer membrane protein	1.39	1.46	1.40	0.83	2.16	0.64
ompQ	outer membrane pyoverdine efflux protein	1.39	1.73	1.40	1.46	1.75	0.74
ompR	two-component system DNA-binding response regulator	1.82	1.52	1.25	0.96	0.92	0.76
opdT-II	Tyrosine-specific outer membrane porin D	1.68	1.55	1.73	1.11	1.31	1.11
oprG	Outer membrane protein OprG	0.99	0.99	1.27	1.32	1.29	1.31
oprH	Outer membrane protein H1	1.24	1.18	1.09	1.16	1.21	1.24

Gene	COG Annotation	Fold Change of FPKM *					
		**Ibuprofen**	**Naproxen**	**Gemfibrozil**	**Diclofenac**	**Propranolol**	**Iopromide**
oprI	Major outer membrane lipoprotein	1.16	0.99	1.48	1.13	1.24	1.34
oprJ	Outer membrane protein OprJ	1.21	1.97	1.36	1.15	1.39	1.12
PP_0143	Membrane protein	0.85	1.18	1.27	1.34	1.35	0.86
PP_0426	Membrane protein	1.06	0.88	1.18	1.27	1.06	1.06
PP_0717	Membrane protein	1.46	1.84	3.34	1.56	1.02	0.72
PP_0828	Membrane protein	2.17	1.78	1.45	0.88	2.95	1.89
PP_0984	Membrane protein	1.91	1.83	1.19	1.39	1.84	2.00
PP_1150	Membrane protein	1.80	1.62	1.34	1.37	1.16	1.01
PP_1159	Membrane protein	2.16	0.58	1.39	1.59	0.74	1.38
PP_1359	Membrane protein	1.15	1.13	2.85	1.88	2.17	1.16
PP_1728	Membrane protein	1.42	1.35	0.83	1.44	1.01	0.59
PP_1936	Membrane protein	1.51	1.66	1.57	1.35	1.97	1.22
PP_2014	Membrane protein	1.47	1.12	1.83	1.22	1.01	1.09
PP_2104	Membrane protein	0.93	1.16	1.51	1.08	1.07	1.16

Gene	COG Annotation	Fold Change of FPKM *					
		Ibuprofen	Naproxen	Gemfibrozil	Diclofenac	Propranolol	Iopromide
PP_2384	Membrane protein	2.19	2.31	--	3.56	--	2.22
PP_2401	Membrane protein	1.27	1.06	--	3.16	2.08	--
PP_2429	Membrane protein	0.95	1.45	1.88	1.78	2.23	1.55
PP_2721	Membrane protein	1.44	12.64	2.77	2.57	2.79	3.14
PP_3105	Membrane protein	1.40	2.33	2.38	1.17	1.39	1.68
PP_3169	Membrane protein	1.09	1.69	1.51	1.11	0.95	2.06
PP_3329	Membrane protein	1.11	0.97	1.30	1.28	1.66	1.53
PP_3389	Membrane protein	1.91	2.17	4.96	1.68	1.30	3.23
PP_3609	Membrane protein	1.46	1.26	2.69	1.18	1.62	0.86
PP_3661	Membrane protein	1.27	1.30	2.45	1.47	1.58	1.80
PP_4118	Membrane protein	1.24	1.34	2.25	1.72	1.16	2.33
PP_4598	Membrane protein	1.01	1.19	1.60	1.95	1.74	2.10
PP_4771	Membrane protein	2.75	3.27	1.91	1.09	0.69	0.57
PP_4815	Membrane protein	1.04	1.32	1.37	1.23	1.15	1.46

Gene	COG Annotation	Fold Change of FPKM *					
		Ibuprofen	Naproxen	Gemfibrozil	Diclofenac	Propranolol	Iopromide
PP_4954	Membrane protein	0.39	0.03	0.52	0.22	-0.11	0.33
PP_5091	Membrane protein	0.07	0.58	1.78	1.11	1.22	1.62
PP_5133	Membrane protein	1.39	-0.27	0.88	0.33	-0.28	0.06
PP_5460	Membrane protein	0.76	0.43	1.08	0.36	-0.25	0.47

*: Comparing with the control group without pharmaceutical dosage

--: not detected

Table S15. Proteins relevant to cell membrane in recipient bacteria *P. putida* KT2440 after exposure of non-antibiotic pharmaceuticals

Protein	Description	Fold Change of Protein Abundance *					
		**Ibuprofen**	**Naproxen**	**Gemfibrozil**	**Diclofenac**	**Propranolol**	**Iopromide**
BamA	Outer membrane protein assembly factor	2.23	1.15	1.78	1.59	1.49	1.95
OmpA	OmpA family protein	1.55	1.28	1.78	2.04	1.11	1.39
OprD	Basic amino acid specific porin	1.59	1.09	1.23	1.01	0.79	1.05
OprE	Outer-membrane porin E	1.35	0.91	1.13	0.79	0.90	1.06
OprG	Outer membrane protein OprG	2.41	1.75	2.00	1.21	0.84	1.30
OprH	Outer membrane protein H1	1.39	0.97	1.24	1.17	1.09	1.08
OprI	Major outer membrane lipoprotein	1.24	1.09	1.44	1.01	0.68	0.72
OprL	Peptidoglycan-associated lipoprotein	1.31	0.95	1.11	0.99	0.97	1.49
OprQ	Outer-membrane porin D	1.69	1.21	1.34	1.37	1.27	1.37
TtgC	Probable efflux pump outer membrane protein TtgC	1.93	1.05	1.77	1.21	1.38	1.58

*: Comparing with the control group without pharmaceutical dosage

Table S16. Genes relevant to conjugative transfer, pilus generation, plasmid replication in IncP-α RP4 plasmid after exposure of non-antibiotic pharmaceuticals

Gene	COG Annotation	Fold Change of FPKM *					
		**Ibuprofen**	**Naproxen**	**Gemfibrozil**	**Diclofenac**	**Propranolol**	**Iopromide**
korB	Global regulator	0.81	0.93	0.59	0.87	0.73	0.93
traG	Conjugative transfer transcriptional regulator	2.14	1.50	0.50	1.01	0.58	0.80
trbD	Conjugative transfer transcriptional regulator	1.87	1.95	2.30	1.13	0.75	0.64
trbA	Mating-pair apparatus	1.94	2.31	1.13	1.29	0.92	0.93
trbK	Mating-pair apparatus	3.43	3.84	2.58	0.74	3.54	0.33
trfA2	Mating-pair apparatus	236.90	133.83	269.91	56.16	147.66	0.07
traC1	Replication regulator	2.01	2.41	1.38	1.35	1.39	1.28
traB	Pilin regulator	1.45	1.22	1.16	1.32	0.90	0.80
traE	Pilin regulator	1.98	1.42	1.23	1.29	0.89	0.99
traF	Pilin regulator	3.25	3.06	1.68	1.24	1.97	0.64
traP	Pilin regulator	1.17	15.44	2.39	1.53	1.60	1.09

*: Comparing with the control group without pharmaceutical dosage

Table S17. Genes relevant to fimbriae in donor bacteria *E. coli* K-12 LE392 after exposure of non-antibiotic pharmaceuticals

**Gene**	**COG Annotation**	**Fold Change of FPKM** *					
		**Ibuprofen**	**Naproxen**	**Gemfibrozil**	**Diclofenac**	**Propranolol**	**Iopromide**
ecpA	ECP pilin	0.70	1.78	4.72	1.69	3.43	1.83
fimH	Minor component of type 1 fimbriae	1.59	1.38	2.77	1.01	1.78	2.07
fliI	Flagellum-specific ATP synthase	0.72	8.17	2.30	3.18	1.36	6.50
hofC	Assembly protein in type IV pilin biogenesis transmembrane protein	0.59	1.12	3.56	1.19	1.56	5.54
yadN	Putative fimbrial-like adhesin protein	3.34	3.71	3.20	3.36	2.87	1.55
ybgO	Putative fimbrial protein	0.95	2.36	1.83	1.69	1.55	0.85
ybgP	Putative periplasmic pilin chaperone	3.14	1.54	7.46	3.29	5.35	1.06
ycbV	Putative fimbrial-like adhesin protein	3.63	4.44	2.36	0.74	6.28	0.73

Gene	COG Annotation	Fold Change of FPKM *					
		Ibuprofen	Naproxen	Gemfibrozil	Diclofenac	Propranolol	Iopromide
yfcQ	Putative fimbrial-like adhesin protein	6.36	4.72	6.50	11.63	1.21	1.21
yfcS	Putative periplasmic pilin chaperone	1.71	1.47	2.01	2.55	0.74	0.82
yqiI	Fimbrial protein	1.06	6.73	5.94	2.14	1.08	0.55
yraH	Putative fimbrial-like adhesin protein	1.75	1.23	4.56	4.35	1.29	1.73
yraI	Putative periplasmic pilin chaperone	1.84	1.93	17.75	2.25	0.90	0.82
yraK	Putative fimbrial-like adhesin protein	1.06	0.59	1.22	1.44	1.65	1.66

*: Comparing with the control group without pharmaceutical dosage

Table S18. Genes relevant to fimbriae in recipient bacteria *P. putida* KT2440 after exposure of non-antibiotic pharmaceuticals

**Gene**	**COG Annotation**	**Fold Change of FPKM** *					
		**Ibuprofen**	**Naproxen**	**Gemfibrozil**	**Diclofenac**	**Propranolol**	**Iopromide**
flgA	Flagella basal body P-ring formation protein	1.31	1.20	2.07	1.68	1.23	1.21
pilE	Type IV pili biogenesis protein	1.15	1.11	2.17	1.09	2.22	2.08
pilH	Twitching motility protein	1.41	1.23	1.61	1.20	1.32	0.99
pilI	Twitching motility protein	1.30	1.05	1.07	1.89	1.66	1.56
pilJ	Twitching motility protein	1.45	1.15	1.66	1.16	1.40	1.24
pilQ	Type IV pili biogenesis protein	1.78	1.11	1.03	1.25	1.06	0.89
pilT	Twitching motility protein	2.50	1.96	2.06	1.64	2.89	1.04
vgrG-II	Type VI secretion system protein	1.55	2.41	1.78	1.33	2.23	1.84
ycgB	Type IV piliation protein	1.24	1.32	1.12	1.37	1.48	1.72
PP_0607	Type IV pili biogenesis protein FimT	1.74	2.41	2.50	1.28	3.92	0.95
PP_1888	Fimbrial protein	1.78	1.92	2.00	1.55	1.01	1.91

Gene	COG Annotation	Fold Change of FPKM *					
		Ibuprofen	Naproxen	Gemfibrozil	Diclofenac	Propranolol	Iopromide
PP_4081	Type IV/VI secretion system protein	1.17	4.32	2.60	0.93	1.74	0.96

*: Comparing with the control group without pharmaceutical dosage

Table S19. Proteins relevant to fimbriae in recipient bacteria *P. putida* KT2440 after exposure of non-antibiotic pharmaceuticals

**Protein**	**Description**	**Fold Change of Protein Abundance** *					
		**Ibuprofen**	**Naproxen**	**Gemfibrozil**	**Diclofenac**	**Propranolol**	**Iopromide**
FliC	Flagellin	1.48	1.09	0.98	1.16	1.46	1.29
Hcp	Type VI secretion system tube protein	1.60	1.39	1.38	1.57	0.99	0.97

*: Comparing with the control group without pharmaceutical dosage

Table S20. Genes relevant to SOS response and universal stress in donor bacteria *E. coli* K-12 LE392 after exposure of non-antibiotic pharmaceuticals

**Gene**	**COG Annotation**	**Fold Change of FPKM** *					
		**Ibuprofen**	**Naproxen**	**Gemfibrozil**	**Diclofenac**	**Propranolol**	**Iopromide**
lexA	Transcriptional repressor of SOS regulon	1.23	1.45	1.08	1.14	1.01	0.85
umuC	Translesion error-prone DNA polymerase V subunit DNA polymerase activity	1.09	1.39	5.46	1.27	1.95	0.90
umuD	Translesion error-prone DNA polymerase V subunit RecA-activated auto-protease	3.36	2.01	1.26	1.53	3.56	1.13
yebG	DNA damage-inducible protein regulated by LexA	1.42	1.03	0.92	1.19	1.05	0.67
yebK	Putative DNA-binding transcriptional regulator	1.64	1.28	1.06	1.28	1.21	0.85
yedK	DUF159 family protein	4.96	10.34	3.39	1.21	0.55	1.04
uspA	Universal stress global response regulator	0.99	2.53	1.88	1.93	2.17	0.81

Gene	COG Annotation	Fold Change of FPKM *					
		Ibuprofen	Naproxen	Gemfibrozil	Diclofenac	Propranolol	Iopromide
uspC	Universal stress protein	0.67	1.56	1.62	1.12	1.27	0.64
uspD	Stress-induced protein	0.55	2.01	1.44	1.36	1.59	1.05
uspE	Stress-induced protein	1.12	2.10	1.61	1.48	1.84	0.94
uspF	Stress-induced protein ATP-binding protein	1.16	1.25	1.27	0.72	1.09	1.21
uspG	Universal stress protein UP12	0.77	1.77	1.55	1.21	1.53	1.09

*: Comparing with the control group without pharmaceutical dosage

Table S21. Proteins relevant to SOS response and universal stress in donor bacteria *E. coli* K-12 LE392 after exposure of non-antibiotic pharmaceuticals

Protein	Description	Fold Change of Protein Abundance *					
		Ibuprofen	Naproxen	Gemfibrozil	Diclofenac	Propranolol	Iopromide
UspG	Universal stress protein UP12	1.48	1.93	1.61	1.36	1.21	0.84

*: Comparing with the control group without pharmaceutical dosage

Table S22. Genes relevant to SOS response and universal stress in recipient bacteria *P. putida* KT2440 after exposure of non-antibiotic pharmaceuticals

Gene	COG Annotation	Fold Change of FPKM *					
		Ibuprofen	Naproxen	Gemfibrozil	Diclofenac	Propranolol	Iopromide
PP_2326	Universal stress protein	1.19	1.43	1.91	1.26	1.57	0.91
PP_3288	Universal stress protein family protein	1.23	1.57	1.55	1.33	1.47	1.83

*: Comparing with the control group without pharmaceutical dosage

Table S23. Proteins relevant to SOS response and universal stress in recipient bacteria *P. putida* KT2440 after exposure of non-antibiotic pharmaceuticals

**Protein**	**Description**	**Fold Change of Protein Abundance** *					
		**Ibuprofen**	**Naproxen**	**Gemfibrozil**	**Diclofenac**	**Propranolol**	**Iopromide**
NP_745431.1	Universal stress protein family protein	1.18	1.08	1.01	1.56	1.01	1.04

*: Comparing with the control group without pharmaceutical dosage

Table S24. Genes relevant to efflux pump and repressor to antibiotic sensitivity in donor bacteria *E. coli* K-12 LE392 after exposure of non-antibiotic pharmaceuticals

Gene	COG Annotation	Fold Change of FPKM *					
		Ibuprofen	Naproxen	Gemfibrozil	Diclofenac	Propranolol	Iopromide
aaeX	DUF1656 family putative inner membrane efflux pump associated protein	4.66	1.16	1.48	2.13	1.26	2.31
mdtJ	Multidrug efflux system transporter	1.85	1.27	1.35	3.71	2.31	2.95
yhiI	Putative membrane fusion protein (MFP) of efflux pump	1.21	1.06	3.68	1.57	1.75	2.22
kdgR	KDG regulon transcriptional repressor	2.00	0.99	1.39	1.20	1.23	1.11

*: Comparing with the control group without pharmaceutical dosage

Table S25. Genes relevant to efflux pump in recipient bacteria *P. putida* KT2440 after exposure of non-antibiotic pharmaceuticals

**Gene**	**COG Annotation**	**Fold Change of FPKM** *					
		**Ibuprofen**	**Naproxen**	**Gemfibrozil**	**Diclofenac**	**Propranolol**	**Iopromide**
czcA-I	Cation efflux system protein	1.68	2.77	1.23	1.37	1.37	1.68
czcA-II	Cation efflux system protein	2.08	1.91	1.22	1.01	0.81	0.78
PP_3789	Efflux transporter	1.16	2.73	2.17	1.26	4.53	1.29
PP_0805	Outer membrane efflux protein	1.09	1.16	2.19	1.31	1.89	0.85
PP_1152	Membrane fusion efflux protein	1.21	3.03	1.07	1.39	1.41	0.61
PP_4923	Outer membrane efflux protein	1.47	0.90	1.44	1.45	1.23	0.85

*: Comparing with the control group without pharmaceutical dosage

References

1 Qiu Z, Shen Z, Qian D, Jin M, Yang D, Wang J*, et al.* Effects of nano-TiO2 on antibiotic resistance transfer mediated by RP4 plasmid. *Nanotoxicology.* 2015; 9: 895-904.

2 Guo J, Gao S-H, Lu J, Bond PL, Verstraete W, Yuan Z. Copper oxide nanoparticles induce lysogenic bacteriophage and metal-resistance genes in Pseudomonas aeruginosa PAO1. *ACS applied materials & interfaces.* 2017; 9: 22298-22307.

3 Wang Y, Lu J, Mao L, Li J, Yuan Z, Bond PL*, et al.* Antiepileptic drug carbamazepine promotes horizontal transfer of plasmid-borne multi-antibiotic resistance genes within and across bacterial genera. *ISME J.* 2018: 509-522.

4 Zhang Y, Gu AZ, He M, Li D, Chen JM. Subinhibitory Concentrations of Disinfectants Promote the Horizontal Transfer of Multidrug Resistance Genes within and across Genera. *Environ Sci Technol.* 2017; 51: 570-580.

5 Chang PH, Juhrend B, Olson TM, Marrs CF, Wigginton KR. Degradation of extracellular antibiotic resistance genes with UV254 treatment. *Environ Sci Technol.* 2017; 51: 6185-6192.
